# Superior Induced Pluripotent Stem Cell Generation through Phactr3-Driven Mechanomodulation of Both Early and Late Phases of Cell Reprogramming

**DOI:** 10.34133/bmr.0025

**Published:** 2024-05-21

**Authors:** Mohammad Mahfuz Chowdhury, Samuel Zimmerman, Hannah Leeson, Christian Maximilian Nefzger, Jessica Cara Mar, Andrew Laslett, Jose Maria Polo, Ernst Wolvetang, Justin John Cooper-White

**Affiliations:** ^1^ Australian Institute of Bioengineering and Nanotechnology (AIBN), The University of Queensland, St. Lucia, QLD 4072, Australia.; ^2^ Albert Einstein College of Medicine, Bronx, NY 10461, USA.; ^3^ Institute of Molecular Bioscience, The University of Queensland, St. Lucia, QLD 4072, Australia.; ^4^ Australian Regenerative Medicine Institute, Monash University, Clayton, VIC 3800, Australia.; ^5^ Department of Anatomy and Developmental Biology, Monash Biomedicine Discovery Institute and the Australian Regenerative Medicine Institute, Monash University, Clayton, VIC 3800, Australia.; ^6^ Adelaide Centre for Epigenetics and the South Australian Immunogenomics Cancer Institute, Faculty of Health and Medical Sciences, The University of Adelaide, Adelaide, SA 5005, Australia.; ^7^ School of Chemical Engineering, Andrew N. Liveris Building, The University of Queensland, St. Lucia, QLD 4072, Australia.

## Abstract

Human cell reprogramming traditionally involves time-intensive, multistage, costly tissue culture polystyrene-based cell culture practices that ultimately produce low numbers of reprogrammed cells of variable quality. Previous studies have shown that very soft 2- and 3-dimensional hydrogel substrates/matrices (of stiffnesses ≤ 1 kPa) can drive ~2× improvements in human cell reprogramming outcomes. Unfortunately, these similarly complex multistage protocols lack intrinsic scalability, and, furthermore, the associated underlying molecular mechanisms remain to be fully elucidated, limiting the potential to further maximize reprogramming outcomes. In screening the largest range of polyacrylamide (pAAm) hydrogels of varying stiffness to date (1 kPa to 1.3 MPa), we have found that a medium stiffness gel (~100 kPa) increased the overall number of reprogrammed cells by up to 10-fold (10×), accelerated reprogramming kinetics, improved both early and late phases of reprogramming, and produced induced pluripotent stem cells (iPSCs) having more naïve characteristics and lower remnant transgene expression, compared to the gold standard tissue culture polystyrene practice. Functionalization of these pAAm hydrogels with poly-l-dopamine enabled, for the first-time, continuous, single-step reprogramming of fibroblasts to iPSCs on hydrogel substrates (noting that even the tissue culture polystyrene practice is a 2-stage process). Comparative RNA sequencing analyses coupled with experimental validation revealed that a novel reprogramming regulator, protein phosphatase and actin regulator 3, up-regulated under the gel condition at a very early time point, was responsible for the observed enhanced reprogramming outcomes. This study provides a novel culture protocol and substrate for continuous hydrogel-based cell reprogramming and previously unattained clarity of the underlying mechanisms via which substrate stiffness modulates reprogramming kinetics and iPSC quality outcomes.

## Introduction

The ability to reprogram somatic cells (e.g., skin fibroblasts) to induced pluripotent stem cells (iPSCs) and derive unlimited numbers of all types of somatic cells (with or without gene editing) from them has created exciting new pathways for developing better disease models for efficient drug screening or achieving patient-specific treatments using cell therapies [1]. However, deriving safe and high-quality iPSC (in terms of tumorigenic and immunogenic potential, genomic, transcriptomic, and epigenetic stability and variability in differentiation capacity) is still a lengthy and costly endeavor, representing a major challenge in transitioning iPSC research into clinical or drug screening applications [2,3].

Somatic cell reprogramming to iPSCs can be divided into 3 phases/stages: the early or initiation stage, the duration of which is 3 day to less than a week and characterized by a mesenchymal-to-epithelial transition (MET); the late/maturation stage, from the end of early phase to the end of reprogramming culture; and the stabilization stage, which covers early through to late passaging of iPSCs [4]. While any improvement at the early stage of reprogramming (entry of somatic cells to reprogramming-prone cells) represents a favorable outcome, improvement in the late stage of reprogramming is crucial, as many reprogramming-prone cells from the early stage fail to establish their pluripotency gene network in the late or maturation phase, thereby reducing the overall reprogramming efficiency [4]. It has been suggested that increasing the kinetics and efficiency of the reprogramming process would also likely positively impact iPSC quality/characteristics and reduce the arduous and expensive tasks of screening for high-quality iPSCs [2].

It is clear that the original somatic cell quality exerts influence over the derived iPSC product and that the reprogramming methodologies and culture conditions used to reprogram cells also influence reprogramming efficiency and iPSC quality [2,3]. Reprogramming efficiency has also been shown to be enhanced by a variety of biochemical-based methods (e.g., mRNA, protein, or small molecules) [5,6]; however, it remains largely unclear whether, and how, biophysical cues presented to cells can improve reprogramming outcomes. Matrix/substrate “stiffness” has been shown to be a key regulator of mechanotransduction pathways impacting adult and pluripotent stem cell behavior, but, to date, our understanding of the impact of substrate stiffness on cell reprogramming outcomes remains limited [7–11]. Choi and coworkers [9] successfully reprogrammed mouse embryonic fibroblasts (MEFs) in a 3-stage process by first plating them onto tissue culture polystyrene (TCPS) for 2 d and transducing them with virus packaging 4 reprogramming factors (Oct4, Sox2, Klf4, and c-Myc), then replating them onto 2-dimensional (2D) polyacrylamide (pAAm) hydrogel substrates of varying stiffness (ranging from 0.1 to 20 kPa) coated with gelatin {using sulfo-SANPAH [sulfosuccinimidyl 6-(4′-azido-2′-nitrophenylamino)hexanoate] (S-S) for surface activation} for 1 day, and thereafter replating them on mitomyocin-C-treated MEF feeder cells on TCPS for the remainder of the reprogramming process (up to 21 day). They observed 2× more mouse iPSCs (miPSCs) generated on TCPS that was inoculated with day 1 (d1) cells exposed the 0.1-kPa substrate, compared to those d1 cells exposed to the 20-kPa substrate. By constructing polyethylene glycol (PEG)-based 3D hydrogels over a very small range of stiffnesses (from 0.3 to 1.2 kPa), Caiazzo and coworkers [11] reported that the physical confinement exerted by the 3D hydrogel on cells induced their earlier entry into MET and epigenetic modification at the early stage of reprogramming that ultimately produced 2.5 and <2 times more miPSC and human iPSC (hiPSC) colonies, respectively, compared to their 2D PEG-based hydrogel controls. Most recently, Kim and coworkers [12] demonstrated small improvements of mouse fibroblast reprogramming to iPSC by introducing already transduced fibroblasts (after 3 d of reprogramming on TCPS) into a 3D methacrylated hyaluronic-acid-based hydrogel, which had a stiffness of 0.15 kPa. In another study, mouse or human somatic cells were first transduced while on TCPS and then, after d4 or d7, replated on a dynamically responsive, very soft (<0.01 kPa) hydrogel to achieve ~8 times (for mouse fibroblast) and 50 times (for human fibroblasts) more iPSC colonies than TCPS [13]. Sun and coworkers [14] reported the generation of hiPSCs from urinal cells, after 1 d of transduction on TCPS, in synthetic 3D peptide hydrogels (*E* < 1 kPa) at equal efficiency to TCPS but with different characteristics. These previous studies have shown that hydrogels of very low stiffness (<1 kPa, ~0.1 kPa) can improve reprogramming outcomes, suggested to be achieved through the modulation of mechanosensitive (e.g., YAP/TAZ) signaling pathways and/or epigenetic changes impacting MET at the early stage of reprogramming. Unfortunately, within all of these studies, direct comparisons with the gold standard practice of using TCPS only throughout the whole reprogramming process were absent, and the actual advantage of these hydrogel substrates/matrices over gold standard practices was not clearly exemplified. In addition, these investigations have all involved multiple steps (effectively multiple passages) in which the cells were exposed for limited amounts of time to the hydrogels either prior to exposure to reprogramming factors or thereafter.

The substrates investigated to date have been significantly lower (by multiple orders of magnitude) in stiffness than TCPS (i.e., ≥10^6^ kPa). Moreover, because of the way in which previous investigations into the impact of substrate stiffness on reprogramming have been performed (as highlighted above), there is little or no information available on the kinetics of the reprogramming process from start to end on substrates of varying stiffness (as no study has achieved such an outcome) and whether these substrates produce iPSC of different characteristics to those produced from TCPS. Such information could be crucial if wishing to combine other biomaterial features (e.g., topography [10]) and soluble factors or small molecules with these substrates to optimize the reprogramming process for producing quality iPSCs at shorter time scale and less cost, facilitating their effective use in regenerative medicine or drug screening.

In this study, we have systematically assessed the extent to which hydrogel-based culture substrates of varying stiffness over ranges of 0.1 to 1,300 kPa modulate the transition of murine and human somatic fibroblasts to iPSCs. Unlike most of the protocols used in investigating reprogramming on different biomaterial substrates [9,10,12], we herein established a consistent gel condition for continuous (single-stage) reprogramming of both human and mouse fibroblasts to iPSCs that did not require fibroblast replating at any time during the reprogramming process. We show how the gel condition impacted the early and the late stages of the reprogramming and overall reprogramming outcomes. Furthermore, by combining reprogramming time-course RNA sequencing (RNA-seq) analysis and experimental observations, we evaluated reprogramming kinetics and the characteristics of iPSCs produced under the gel and TCPS conditions and identified a novel and critical regulator of the observed improvements.

## Materials and Methods

### pAAm hydrogel fabrication

Coverslips were cleaned in a Harrick Plasma Cleaner (PDC-002-HP, 45 W) at 600 μm for 10 min. They were then soaked in prepared 0.4% (v/v) of 3-(trimethoxysilyl)propyl methacrylate) (M6514, Sigma-Aldrich) in distilled H_2_O (dH_2_O; pH balanced to 3.5 using glacial acetic acid) for 1 h, before being washed twice with dH_2_O and dried in an oven at 120 °C for 1 h. To increase gel stiffness from 1 to 247 kPa, 40% (m/v) of acrylamide (AAm) (A3553, Sigma-Aldrich) and 2% (m/v) of bis-AAm (M7279, Sigma-Aldrich) stock solutions were mixed with dH_2_O to achieve the desired percentage (m/v) of AAm and corresponding percentage (m/v) of bis-AAm by maintaining their ratio at 29:1. For pAAm gel of 1.3 MPa, 180% AAm and 5% bis-AAm solutions were mixed to achieve 80% (m/v) of AAm and corresponding percentage (m/v) of bis-AAm. To form the gel, 4 μl of tetramethylethylenediamine (T9281, Sigma-Aldrich) and 6 μl of ammonium persulfate (10%, m/v; A3678, Sigma-Aldrich) in dH_2_O were also added to AAm and bis-AAm solution. Then, 150-μl drops of the complete solution were distributed on a hydrophobic glass slide [hydrophobized using Sigmacote (SL2, Sigma-Aldrich)]. The activated coverslips were then overlaid onto the drops, and polymerization was continued for 20 to 30 min. Coverslips with the gels were gently dislodged and washed twice with phosphate-buffered saline (PBS) and stored at 4 °C.

### Rheology of the gels

For oscillatory rheology, gels were formed over nonactivated coverslips by distributing 300 μl of gel solution. Gels were peeled off from the coverslips and placed between the plates (top plate diameter, 8 mm) of an oscillatory rheometer (Discovery HR-2, TA Instruments). Gel excess was scraped off using a scalpel, and the top plate was lowered until a small bulge around the gel was observed. Three microliters of PBS was distributed around the gel before starting each rheological measurement to keep the gel hydrated.

For rheological measurements, strain sweeps using strain range (0.01% or 10%) at a frequency of 10 rad/s were performed to obtain the Storage (*G*’) and loss (*G*”) modulus versus strain curve. *G*’ and *G*” were then determined at various frequencies (range of 1 to 100 rad/s) using the specific strain value obtained from the linear viscoelastic range of the curve. Finally, *G*’ and *G*” values at 10 rad/s were reported as the gel’s rheological properties. From these values, the Young’s modulus (*E*) was calculated using *E* = 2*G*(1 + ν), where *G* = ((*G*’)^2^ + (*G*”)^2^)^½^ and *ν* is Poisson’s ratio of 0.457 [15].

To compare oscillatory rheometer measurements with stiffnesses measured by atomic force microscopy (AFM), hydrogels made with 20% AAm stiffness was measured with an MFP-3D AFM (Oxford Instruments). For AFM, samples were loaded on a glass slide; a drop of PBS was placed on the sample. All experiments were performed in PBS at an ambient temperature. Force maps using SiNi cantilever (0.27 N/m) were performed at 20 nN of load and at a velocity of 1 μm/s. The hertz model was applied on each force curve obtained in the force map to calculate the Young’s modulus. The values are displayed with SD.

### Extracellular matrix immobilization using S-S and 3,4-dihydroxy-l-phenylalanine

Extracellular matrix (ECM) immobilization with S-S treatment was performed according to a published protocol [16]. Briefly, 50 mg of S-S (catalog no. 22589, Thermo Fisher Scientific) was dissolved in 1 ml of dimethyl sulfoxide (DMSO) and diluted 25 times in Milli-Q water, resulting in an S-S solution (2 mg/ml). Three hundred microliters of this solution was added to the gel surface and placed under an ultraviolet light in a biological safety cabinet for 30 min. Gels were then washed twice with PBS and incubated with the desired ECM (0.2% gelatin in PBS; G1890, Sigma-Aldrich) overnight at 4 °C.

Gels were prepared for cell culture by washing once in PBS, rinsing in 70% ethanol for 2 min, washing in PBS twice, ultraviolet-sterilizing for 30 min, and incubating with culture medium for 1 h. For ECM conjugation using L-DOPA (3,4-dihydroxy-l-phenylalanine; D9628, Sigma-Aldrich) [17], L-DOPA solution (2 mg/ml) was prepared using 10 mM tris-HCl buffer (pH balanced to 10.2 using 1 M NaOH) by mixing gently on a roller shaker for 30 min under no-light conditions. The solution was filtered to remove undissolved L-DOPA, distributed over the gels, and then incubated for 30 min under no-light conditions. Gels were then conjugated with ECM and prepared for cell culture as per the procedure described for S-S above.

### MEF reprogramming

MEF cultures containing doxycycline (Dox)-inducible O (Oct4) K (Klf4) S (Sox2) M (Myc), i.e., OKSM cassette, and endogenous Oct4-driven green fluorescent protein (GFP) expression locus were provided by Polo group [18] at Monash University. MEFs were expanded for one passage in MEF medium [high-glucose Dulbecco’s modified Eagle’s medium, 10% fetal bovine serum (FBS), sodium pyruvate, l-glutamine, penicillin–streptomycin, nonessential amino acids, and 2-mercaptoethanol) for preparing cell stocks in FBS with 10% DMSO. These MEFs (inoculated at 25,000 cells/cm^2^) were expanded for 3 d maintaining <90% confluency before inoculating them for reprogramming at 2,000 cells/cm^2^ on the gel or TCPS conditions in embryonic stem cell (ESC) medium [knockout DMEM with 15% FBS, l-glutamine, penicillin–streptomycin, nonessential amino acids, b-mercaptoethanol, and leukemia inhibitory factor (1,000 U/ml)] and Dox (2 μg/ml; catalog no. 33429, Sigma-Aldrich). MEFs were reprogrammed in the presence of Dox for 12 day and without Dox for the past 5 day. Medium was changed every other day. At the end the reprogramming, Oct4 GFP^+^ iPSC colonies in each well or hydrogel on a coverslip were counted under fluorescence microscope, and Oct4 GFP^+^ cells were quantified by flow cytometry.

### Human fibroblast reprogramming

To validate the efficacy of 102 kPa pAAm gel to improve human cell reprogramming, we utilized a modified Sendai-based transfection protocol to reprogram human dermal neonatal foreskin fibroblasts (hDFns) [19]. In the protocol, hDFns were inoculated at low density (4,000 cells/cm^2 ^) on the substrate and incubated overnight before transfecting with Sendai, and the reprogramming culture was continued without replating for 18 d. We confirmed that transfection of cells at low density with Sendai did not affect cell viability (Fig. S5B). Two different lots (lots 1355434 and 1767629) of hDFns were purchased from Thermo Fisher Scientific (catalog no. C0045C) and expanded twice according to the manufacturer’s instructions. These hDFns (inoculated at 35,000 cm^−2^) were grown for 3 day (maintaining <90% confluency) in MEF medium before inoculating them for reprogramming at 4,000 cells/cm^2^ on the gels or TCPS.

For human cell reprogramming, gels or TCPSs were coated with vitronectin (0.2 μg/cm^2^; A14700, Thermo Fisher Scientific) instead of gelatin. After overnight incubation, cells were transduced with Sendai virus (CytoTune-iPS 2.0 Sendai Reprogramming Kit, A16517, Thermo Fisher Scientific) using multiplicity of infections (MOIs) 5, 5, and 3 (i.e., KOS MOI = 5, hc-Myc MOI = 5, and hKlf4 MOI = 6) in MEF medium. After 24 h (d1), culture medium was exchanged with E8 medium (catalog no. A1517001, Thermo Fisher Scientific). Culture medium was exchanged again on d2 and every other day after that. From d2 to d6, reprogramming was continued in E8 medium and from d6 to d18 in E7 medium [E6 medium, catalog no. A1516401, Thermo Fisher Scientific + basic fibroblast growth factor (Fgf) (100 ng/ml), catalog no. 100-18B, PeproTech]. On d18, the reprogrammed culture was stained for alkaline phosphatase (ALP) activity. ALP^+^ hiPSC colonies in each well or hydrogel on a coverslip were counted, and SSEA-4^+^/TRA-1-60^+^ hiPSCs were quantified by flow cytometry.

### Cell counting

Cells were dissociated to singe cells with Trypsin-EDTA (catalog no. 25200056, Thermo Fisher Scientific) or TrypLE (catalog no. 12604013, Thermo Fisher Scientific) according to the manufacturer’s instructions. Fifty microliters of the single cell suspension was mixed with equal volume of Trypan Blue (catalog no. 15250061, Thermo Fisher Scientific), and the final solution was used to count cells automatically using TC20 automated cell counter (Bio-Rad) according to the manufacturer’s instruction.

### ALP staining

Cells were fixed with 4% paraformaldehyde solution for 15 min and washed once with PBS. Afterward, a filtered solution containing Napthol AS-MX phosphate disodium salt (0.2 mg/ml; Sigma-Aldrich) and Fast Red TR salt hemi (zinc chloride) salt (1 mg/ml) in 100 mM tris-HCl buffer (pH 9.2) was distributed over the cells and incubated for 20min. Finally, cells were washed twice with PBS.

### Flow cytometry

Mouse cells and human cells were dissociated with 0.25% Trypsin-EDTA (catalog no. 25200056, Thermo Fisher Scientific) and TrypLE (catalog no. 12604013, Thermo Fisher Scientific), respectively. Dissociated cells were washed once with 1% bovine serum albumin (BSA) in PBS before incubating with primary antibodies for 30 min. The following primary antibodies were used for mouse and human cells: anti-Thy1.2 phycoerythrin (PE)-cyanine (1:400; catalog no. 25-0902, eBioscience), anti-Ssea-1 biotin (1:400; catalog no. 13-8813, eBioscience), anti-CD13 PE-cyanine (2.5 μl per test; catalog no. 561599, BD Pharmingen), anti-SSEA-4 PE (2.5 μl per test; catalog no. 330406, BioLegend), and anti-TRA-1-60 Alexa Fluor 647 (2.5 μl per test; catalog no. 330606, BioLegend). Cells were washed twice with 1% BSA in PBS and incubated with the secondary antibody streptavidin APC (1:200; catalog no. 17-4317, eBioscience) for 30 min, if necessary. Finally, cells were washed twice in 1% BSA in PBS containing propidium iodide and passed through a 40-μm cell strainer to achieve single-cell suspension. Cells were analyzed in a LSRII (BD Biosciences) and sorted by Influx cell sorter instrument (BD Biosciences)**.**

### RNA analysis

Total RNA isolation from cell pellets was performed according to the manufacturer’s instructions for the RNeasy Mini or Micro Kit (catalog no. 74104 or 74004, QIAGEN). RNA was eluted from the columns using 20 μl of ribonuclease-free water and quantified using NanoDrop.

### RNA sequencing

Total RNA samples were sequenced on HiSeq 1500 (Illumina Inc.) using HiSeqV2 rapid chemistry according to the manufacturer’s instructions in Monash Health Translation Precinct RNA-seq facility. All RNA-seq data will be available from the Gene Expression Omnibus repository.

### Bioinformatics analysis

#### RNA-seq raw data processing

Reads were aligned using the custom genome and gtf file (gencode vM4) provided by Polo et al. [18] with the STAR aligner in per-sample 2-pass mapping mode (version 2.5.1b). The number of reads maps to each gene was counted using htseq-count248 (version 0.6.1) with Python version 2.7.8. Since our RNA-seq data are from a strand specific assay, the stranded parameter was set to reverse. The order parameter was also set to “pos” since the alignment was sorted by alignment position. Count data were converted to RPKM (reads per kilobase million) values using the function “rpkm” from edgeR (version 3.18.1 and limma version 3.32.5) with norm.lib.sizes set to TRUE (default). The lengths of each gene were acquired using featureCounts on one sample and the custom gtf file. The RPKM values were log_2_-transformed with one added to each value to prevent NAs (not availables).

#### Enrichment analysis of MIKKELSEN gene sets in gene set enrichment analysis

To perform enrichment of various MIKKELSEN gene sets [20] in gene set enrichment analysis (GSEA), which contain differentially regulated or methylated genes in MEF, mouse ESC (mESC), and fully and partially reprogrammed cells, C2-curated gene sets for mouse were downloaded from http://bioinf.wehi.edu.au/software/MSigDB/, and a camera test was performed in R using the gene sets and differentially expressed genes (DEGs) between Oct4 GFP^+^ cells in TCPS and pAAm. Although iPSC (passage 0) generated from TCPS and pAAm gel clustered quite closely, over 3,000 genes [false discovery rate (FDR) = 0.05] were differentially expressed between them at d17.

Although MIKKELSEN_PLURIPOTENT_STATE_UP/DOWN (genes up/down-regulated in the iPSC and ESC compared to the parental lineage-committed and partially reprogrammed cell lines) regulation was not different (FDR > 0.05), MIKKELSEN_DEDIFFERENTIATED_STATE_UP and DOWN (genes up/down-regulated in partially reprogrammed, iPSC and ESC compared to parental lineage-committed cell lines) were up- and down-regulated in iPSCs produced on pAAm gels compared to TCPS (Fig. S6A). Along with the down-regulation of gene sets that are inactive in ESC and iPSC (marked by H3K27ME3 and both H3K4ME3 and H3K27ME3), iPSC under the pAAm condition down-regulated gene sets active in MEF (MIKKELSEN_MEF_LCP_WITH_H3K4ME3) compared to TCPS (Fig. S6A).

#### Kyoto Encyclopedia of Genes and Genomes pathway analysis

Kyoto Encyclopedia of Genes and Genomes (KEGG) pathway analysis was performed using kegga function in limma (v.3.28.14) available in R. FDR < 0.05 and *P* < 0.05 were used to filter DEGs and differentially regulated pathways, respectively.

#### Principal components analysis

Principal components analysis (PCA) plot was generated using plotPCA in DESeq2 in R (version 3.4.1).

#### Identification of molecules accelerating reprogramming in pAAm gel

First, we performed STRING protein–protein interaction network analysis of the DEGs (FDR < 0.05 and log fold change > 1.5) at d3 by classifying them under various Gene Ontology (GO) terms: focal adhesion, cytoskeleton organization, cellular response to hypoxia, tight junction, transforming growth factor-β signaling pathway, stem cell population maintenance, epithelium development, nucleus, and metabolic process (Fig. S8) using Cytoscape (v3.6.1). These GO terms were relevant to the essential up/down-regulated KEGG pathways at d3 identified by GAGE (Generally Applicable Gene Set Enrichment) analysis.

It is well known that when cells are placed on a substrate, they probe the surface by forming and dislodging focal adhesions and adapt with the mechanical environment by changing cytoskeleton organization and ECM [21]. Indeed, cells on pAAm undergo large changes in cytoskeleton organization indicated by down-regulation of various actin binding proteins [22], e.g., Actn1, Cap2, and Tpm2 (Figs. S9 and S10).

Cells on pAAm express higher Fgf2 compared to TCPS, which was strongly associated with lower Fn1 and collagen expression (except collagen 15 and collagen 6) (Figs. S9 and S10). Interestingly, collagen 15 and collagen 6 are the nonfibrillar collagens that are presents in epithelia basal lamina and knockdown of collagen 6 and associated up-regulation of Fn1 caused loss of epithelia cell shape [23,24]. Fgf2 has the potential to induce reorganization and disruption of actin cytoskeleton through phosphatidylinositol 3-kinase, Rho and Cdc42 and reduce the Fn1 expression and Fn1-mediated collagen 1 family expression [25–27]. Jiao and coworkers [28] showed that collagen down-regulation in the presence of Fgf2 at the early stage of reprogramming improved cell reprogramming outcomes, which presumably played a major role in improving cell reprogramming outcome under the pAAm condition in this work.

Interestingly, we observed that Gata2 expression was up-regulated across the whole reprogramming period in pAAm compared to TCPS (Figs. S9 and S10). Arhgap proteins (Rho guanosine triphosphatase activating protein) activate the RhoA pathway to modulate cytoskeletal organization. Various Arhgap proteins (Arhgap22 and Arhgap23) were down-regulated under the pAAm condition on d3, and like Arhgap35, down-regulation of these might have induced up-regulation and translocalization of Gata2 for cells on pAAm [29]. Recently, the Gata transcription factor (TF) family has been shown to be similarly effective as or better than Oct4 to induce reprogramming [30]. Notably, among Gata TFs, Gata2 showed more effectiveness to improve reprogramming [30]. Therefore, besides Fgf and bone morphogenetic protein (Bmp) signaling pathways, Gata2 presumably played an important role in the reprogramming outcomes observed under the pAAm gel condition.

### Knockdown of phosphatase and actin regulator 3 with small interfering RNA

Small interfering RNA (siRNA) against protein phosphatase and actin regulator 3 (Phactr3) or mock scramble siRNA at 4 nM was prepared in Opti-MEM I Reduced Serum Medium (51985-026, Life Technologies) with 16 μl Lipofectamine RNAiMAX Transfection Reagent (13778075, Life Technologies)/ml of solution and incubating the solution at room temperature for 20 min. Then, 1 ml of this solution was mixed with 3 ml of MEF culture medium, and on d1 of routine culture of MEF, at 40% to 50% confluency in T25 flasks, cells were incubated with 4 ml containing siRNA against Phactr3 [ON-TARGETplus mouse Phactr3 (74189) siRNA—SMARTpool, lot-046898-01-0005, Dharmacon) or mock scramble siRNA (DHA-D-001810-10-05 ON-TARGETplus nontargeting pool) at 2 nM and cultured for another 2 day before using them in reprogramming experiments. At the start of reprogramming experiments, MEFs were inoculated on TCPS or pAAm hydrogel in MEF medium without siRNA. On the following day, medium was changed to reprogramming medium containing target siRNA or scramble siRNA. On d2, cells were transfected with respective siRNAs for another 2 day, and, after that, cells were reprogrammed as usual.

### Immunostaining

Cells were rinsed twice in PBS, fixed in 4% paraformaldehyde for 20 min, washed in PBS twice for 5 min each wash, permeabilized with 0.2% Triton X-100 for 10 min, washed again twice in PBS, blocked with 5% goat serum in PBS for 30 min, and incubated with Phactr3 antibody (PA5-65682; 2 μg/ml) overnight. Cells were then washed thrice in PBS, incubated with secondary antibody (4 μg/ml) and/or Alexa Fluor 488 Phalloidin (A12379; 400 times diluted) for 90 min, washed thrice in PBS, stained with 4′,6-diamidino-2-phenylindole (2 μg/ml) for 5 min, and rinsed thrice in PBS. Cells were visualized within 3 d with normal fluorescence or confocal microscopy.

### Quantitative polymerase chain reaction

Total RNA and cDNA were synthesized from the samples using the RNeasy Mini Kit and QuantiTect Reverse Transcription Kit according to the manufacturer’s instructions (QIAGEN). Quantitative polymerase chain reaction (qPCR) was performed in a Bio-Rad thermocycler with a reaction volume of 20 μl that constitutes primers (0.5 μM), cDNA (equivalent to 20 ng of RNA), and PowerUp SYBR Green Master Mix. Cycling condition was 50 °C for 2 min, 95 °C for 2 min, 95 °C for 15 s, and 60 °C for 1 min. The last 2 heating steps were repeated 45 times. Primers are available upon request.

For better visualization of the comparison between TCPS and hydrogel, we transformed all (if not stated otherwise) qPCR data that made TCPS means equal to zero. For the transformation, (a) *C*_T_ values of technical replicates were averaged, and Δ*C*_T_ was calculated by subtracting reference gene (i.e., housekeeping gene) *C*_T_ from gene of interest *C*_T_; (b) for each gene of interest, average Δ*C*_T_ in TCPS was calculated for each gene of interest, and it was subtracted from Δ*C*_T_ of that gene for TCPS and 102 kPa to calculate −ΔΔ*C*_T_, which is equal to log_2_ transformation of 2^−ΔΔ*C*_T_^. Statistical analysis was performed on log_2_-transformed data.

### Statistical analysis

Data were log_2_-transformed before any statistical analysis so that the assumptions of statistical analysis were met [31]. Before the transformation, if the original data contained zero values, one was added to all data to avoid undefined values after transformation. The normality of any dataset was checked with a Shapiro–Wilk test and QQ (quantile–quantile plot) plot. As a *t* test and analysis of variance (ANOVA) are robust measures to manage mild deviations from normal distributions, 0.01 significance level was chosen for the normality test. Comparisons between the means of 2 groups was made with an unpaired 2-tailed *t* test with or without Welch’s correction depending on the *F* test outcome that compared variance equality of the groups. Comparison among means of more than 2 groups was made with ordinary or Brown–Forsythe and Welch one-way ANOVA depending on the Brown–Forsythe test outcome that compared variance equality of the groups. After ANOVA analysis, subsequent multiple comparison of the groups with respect to a control (specified in each figure legend) was made with a Dunnett (in case of equal variances among groups) or a Dunnett T3 (unequal variances among groups) test. All values are expressed as the means ± SEM/SD (as indicated in the figures’ legends). For multiple comparisons, *P* < 0.05 was considered statistically significant. GraphPad Prism (GraphPad Software Inc., San Diego, CA, USA) was used for these analyses.

## Results

### Optimal MEF reprogramming outcomes observed on 102 kPa L-DOPA-treated pAAm hydrogels

To investigate the role of substrate stiffness on cell reprogramming, we seeded MEFs with Dox-inducible OKSM reprogramming factors and an Oct4 GFP reporter on pAAm gels of varying elasticity (*E* from 1 to 1.3 MPa; Fig. S1) with gelatin (0.2%, w/v) that was immobilized on the surface with the S-S conjugation method and assessed reprogramming efficiency into miPSCs by enumerating GFP expressing colonies. This revealed that across the range of gels examined, MEF reprogramming on 102 and 247 kPa gel (Fig. S2A) respectively produced 4- and 3-fold higher numbers of Oct4 GFP^+^ miPSC colonies compared to gelatin-coated TCPS. In addition, 1.3 MPa showed a tendency to improve reprogramming outcomes. However, these S-S-treated gels were unable to support long term culture, as indicated by frequent detachment of cell layer from the culture substrate (Fig. S2B).

We hypothesized that this was due to suboptimal immobilization of gelatin on these pAAm substrates with the S-S method [32], leading us to examine an alternate conjugation method, using L-DOPA [17]. Satisfyingly, the L-DOPA conjugation method produced a more homogenous ECM coating than that achieved with S-S treatment (Fig. S3), and we confirmed that equal amounts of gelatin were deposited on the pAAm gels of varying stiffness and TCPS treated with L-DOPA (Fig. S3).

**Fig. 1.**
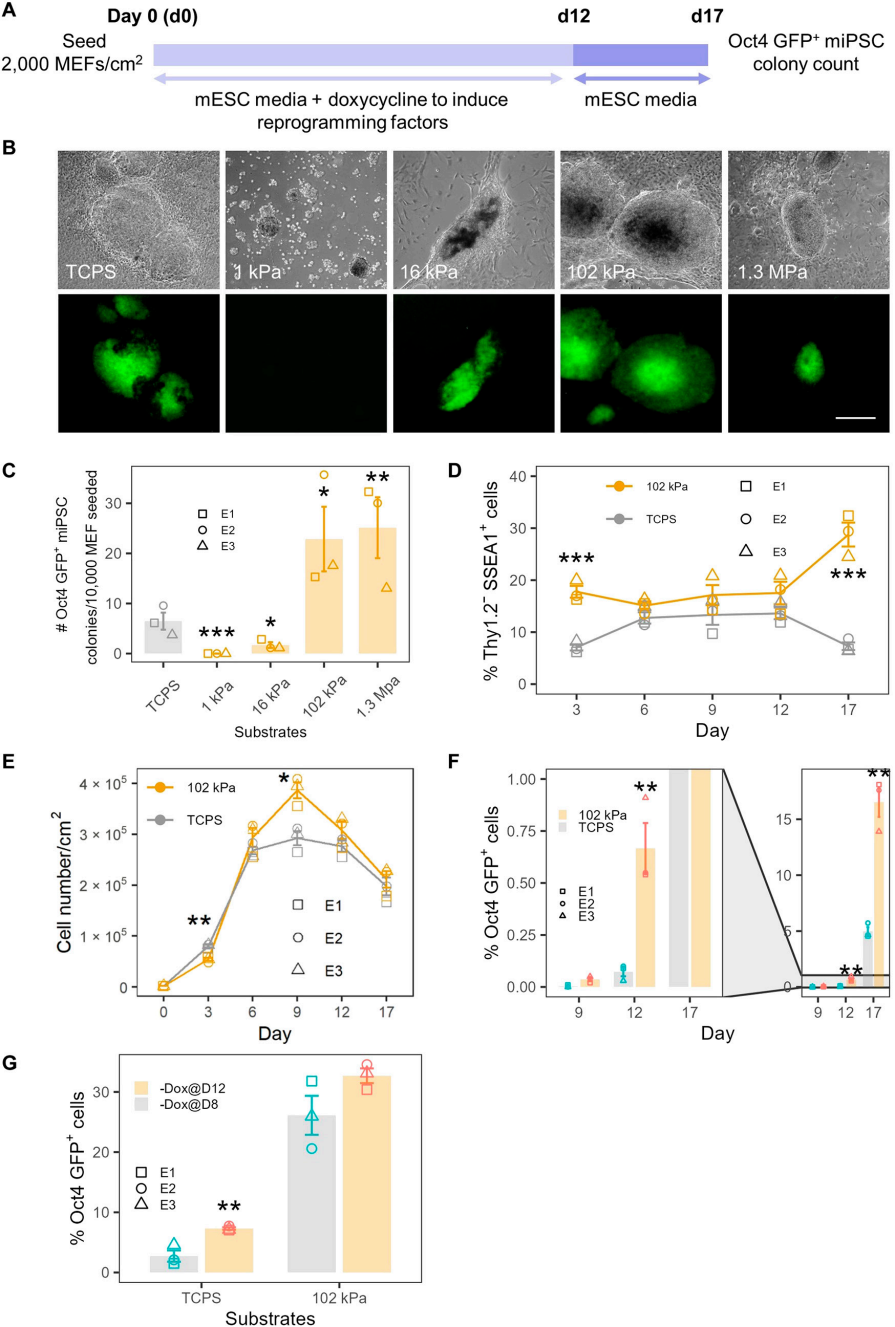
MEF reprogramming on pAAm gels of various stiffnesses, which were conjugated with L-DOPA for ECM immobilization. (A) Summary schematic of reprogramming protocol as described in Materials and Methods. (B) Bright-field (top row) and corresponding GFP channel (bottom row) showing miPSC colonies on TCPS and pAAm gels on d17. Scale bar, 500 μm. (C) Number of Oct4 GFP^+^ miPSC colonies (scaled to per 10,000 MEFs seeded on d0) counted under fluorescence microscope at d17 on TCPS and pAAm gel of various stiffnesses. (D) Percentage of cells undergoing reprogramming (Thy1.2^−^Ssea-1^+^ cells) on TCPS and 102-kPa hydrogel at various days analyzed with flow cytometry. (E) Cell number count on each day, over the reprogramming period in TCPS and pAAm. (F) Emergence of Oct4 GFP^+^ (quantified by flow cytometry) in reprogramming culture on TCPS and pAAm overtime starting from d9. (G) Percentage of Oct4 GFP^+^ miPSCs (quantified by flow cytometry) at d17 on TCPS and pAAm gel after Dox was withdrawn earlier (d8) than the normal (d12). MEFs from 3 embryos (E1, E2 and E3; *n* = 3) were used for reprogramming. Each data point reflects mean of 2 technical replicates (i.e., 2 wells or hydrogel on 2 coverslips) for each MEF line. Error bars represent SEM, and ***P < 0.001, ***P < 0.01, and *P < 0.05 compared to TCPS.

On the basis of MEF reprogramming outcomes in S-S-treated gels, we selected hydrogel stiffnesses that were approximately separated by one order of magnitude in a series (i.e., 1, 16, 102, and 1,300 kPa). When MEFs were reprogrammed on L-DOPA-treated pAAm gels with range of moduli, we found that pAAm gels of *E* = 102 kPa and 1.3 MPa consistently produced 4-fold more Oct4 GFP^+^ colonies than the TCPS control (Fig. 1B and C). In contrast, pAAm gels of 1 and 16 kPa showed very low numbers of Oct4 GFP^+^ colonies compared to the TCPS control (Fig. 1B and C).

Since the reprogramming of MEFs into iPSC consists of 3 separate stages characterized by an initial down-regulation of the fibroblast-associated marker Thy1.2 (d1 to d3), activation of the Ssea-1 antigen (d3 to d9) and eventually up-regulation of the Oct4 GFP reporter (d9 to d17), we were interested to find out which phase of the reprogramming process was affected by substrate stiffness. Since cell reprogramming is intimately connected to cell cycle progression, we first examined cell proliferation on the pAAm gels of varying stiffness, revealing that cell proliferation on all pAAm gels by d3 was reduced as compared to TCPS controls (Fig. S4A). Nevertheless, all pAAm gels produced higher numbers of Thy1.2^−^/Ssea-1^+^ cells compared to the control TCPS on d3 of reprogramming (Fig. S4B). However, the 1 kPa hydrogel was unable to produce miPSC colonies and the 16 kPa gel produced lower number of miPSC colonies compared to TCPS (Fig. 1C), suggesting that these substrates did not support later stages of reprogramming.

Although the 1.3 MPa hydrogel produced more miPSC colonies than TCPS, at a near equal levels to 102 kPa, we noticed both the delayed emergence of Oct4 GFP^+^ miPSC colonies (qualitative observations during reprogramming) and smaller sizes of the colonies (Fig. 1B) in 1.3 MPa gels compared to 102 kPa gels. Moreover, with the 1.3 MPa gels, we observed frequent detachment of the gels from the coverslips that introduced loss of samples over the course of experiment and thus a lack of robustness as a platform. More importantly, regardless of the conjugation method used, only the 102 kPa gel consistently produced higher numbers of iPSC colonies compared to the TCPS. Therefore, we concluded that, of the discrete gel stiffnesses screened, a 102 kPa gel provided the optimal condition for achieving consistent improved reprogramming outcomes, and we thus focused on examining the cell reprogramming process on the 102 kPa gel in detail.

At d3, the percentage of Thy1.2^−^/Ssea-1^+^ cell population was 3-fold higher on the 102 kPa pAAm gel compared to TCPS control (Fig. 1D). Between d6 and d12, the number of Thy1.2^−^/Ssea-1^+^ cells on either the 102-kPa gel or TCPS did not substantially change. However, from d12 to d17, we observed a robust increase in the number of Thy1.2^−^/Ssea-1^+^ cells on pAAm as compared to TCPS, which showed a decrease (Fig. 1D) in Thy1.2^−^/Ssea-1^+^ cells. Although cell growth was noted to decrease in pAAm by d3, it gradually increased during d3 to d9 and maintained a higher cell growth trend (nonsignificant) over the remaining reprogramming period (Fig. 1E).

Between d9 and d17, the 102 kPa gels fostered substantial increases in the number of Oct4 GFP^+^ cells as compared to TCPS (Fig. 1F), indicating that substrate stiffness accelerated the transition of Thy1.2^−^/Ssea-1^+^ cell into pluripotent Thy1.2^−^/Ssea-1^+^/Oct4 GFP^+^ miPSCs. As expected, the removal of Dox on d8 from cells cultured on TCPS resulted in a significant reduction in GFP^+^ cells (Fig. 1G). In contrast, removal of Dox on d8 from cells reprogrammed under the 102 kPa pAAm gels condition did not affect the number of GFP^+^ cells, suggesting that the mechanics of the hydrogel substrate enhanced entry into pluripotency at the early stage and can independently drive the improvement of reprogramming, irrespective of the modulation of reprogramming at the late phase.

Overall, these data indicated that reprogramming of MEFs on a pAAm hydrogel substrate of ~102 kPa leads to a 4-fold increase in Oct4 GFP^+^ miPSCs compared to TCPS by enhancing the number of Thy1.2^−^/SSEA1^+^ cells during the initial phase of reprogramming (d0 to d3) and by fostering the maturation of the pluripotent state during the final reprogramming phase (d9 to d17).

### Significantly enhanced human fibroblast reprogramming outcomes on optimized hydrogel substrate

**Fig. 2.**
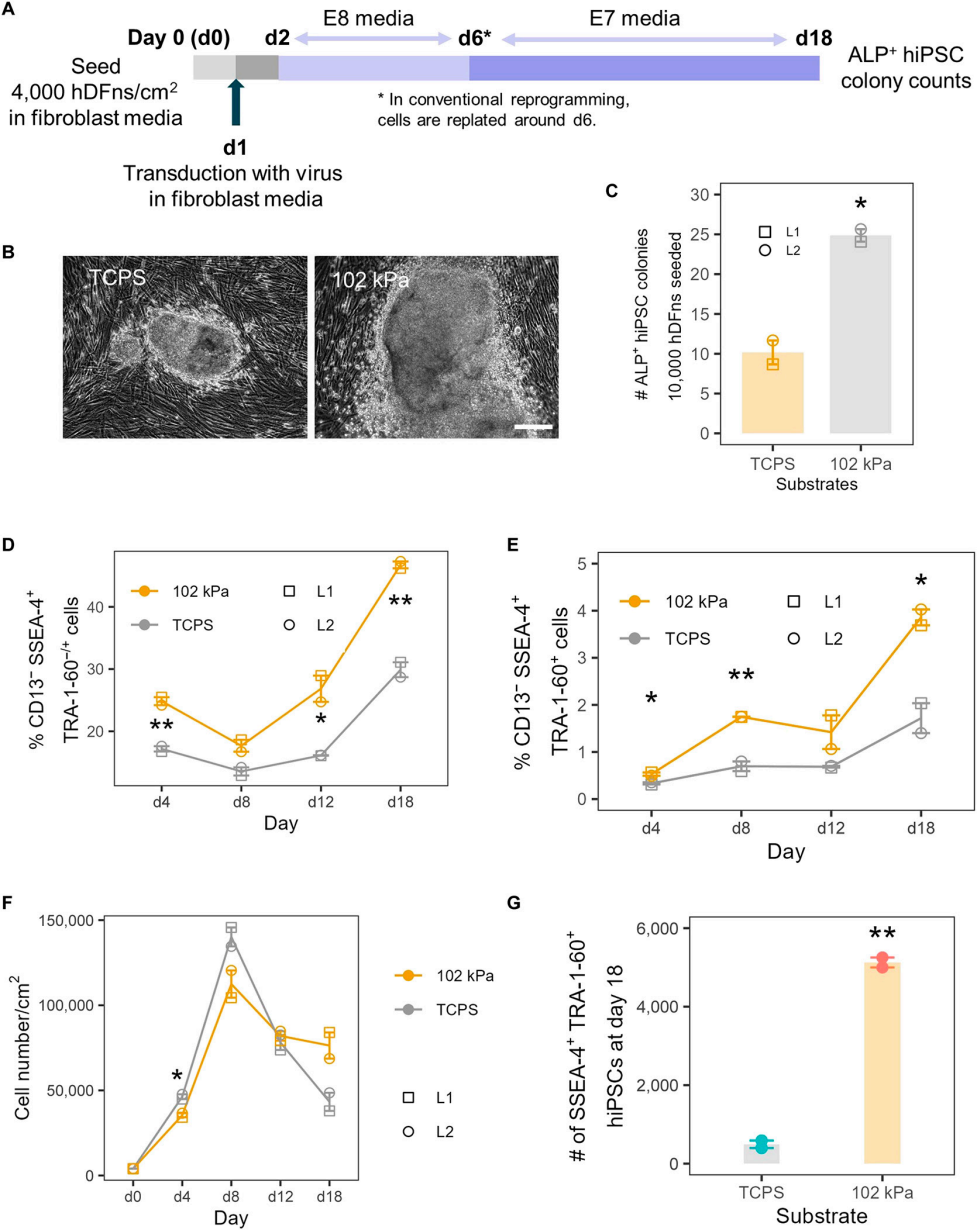
Comparison of hDFns reprogramming on 102 kPa pAAm gel and TCPS. (A) Summary schematic of reprogramming protocol as described in Materials and Methods. (B) Bright-field images of hiPSC colony and (C) number of hiPSC colonies (per 10,000 hDFns seeded for reprogramming) that were positively stained for ALP activity (ALP^+^) at d18 in TCPS and pAAm gel. Scale bar, 500 μm. Percentage of (D) reprogramming prone CD13^−^SSEA-4^+^TRA-1-60^−/+^ cells and (E) percentage (in log_2_ scale for better data point visualization) of bona fide reprogrammed CD13^−^SSEA-4^+^TRA-1-60^+^ cells over time in TCPS and pAAm gel quantified by flow cytometry. (F) Cell growth (quantified by cell number) over time in TCPS and pAAm. (G) Yield (number of iPSC per 10,000 inoculated hDFn at d0) of hiPSC at d18. Two hDFn lines (L1 and L2; *n* = 2) were used for reprogramming. Each data point reflects mean of 2 technical replicates (i.e., 2 wells or hydrogel on 2 coverslips) for each hDFn line. Error bars represent SEM, and **P < 0.01 and *P < 0.05 compared to TCPS.

We next wished to assess whether 102 kPa pAAm gels would also enhance Sendai-virus-mediated reprogramming of hDFns. To avoid the replating of cells, a process that is customary in the standard Sendai virus protocol on TCPS, we used lower fibroblast seeding densities than usual, allowing us to assess the effect of substrate stiffness on human fibroblast reprogramming to hiPSC from the start to the end (Fig. 2A).

The 102 kPa pAAm gel condition produced 2 times higher ALP^+^ hiPSC colonies compared to TCPS (Fig. 2B and C and Fig. S5A) at d18, demonstrating for the first time the efficacy of 102 kPa pAAm gel in improving human cell reprogramming. Time-course profiles of human cells undergoing reprogramming (using marker panels reported in [33]) revealed: an increase in CD13^−^SSEA-4^+^TRA-1-60^+/−^ cells at earlier time points (d4), relatively little increases during the middle period (d8 to d12), and a large increase in fully reprogrammed CD13^−^SSEA-4^+^TRA-1-60^+^ cells during the late reprogramming period (d12 to d18) (Fig. 2D and E). The 102-kPa gel condition produced a modest increase (2 times) in the reprogramming-prone (SSEA-4^+^TRA-1-60^+/−^) populations compared to TCPS but produced 6 times higher amounts of fully reprogrammed hiPSCs (CD13^−^SSEA-4^+^TRA-1-60^+^) at d18 (Fig. 2E), again indicating that the 102 kPa hydrogel efficiently fostered entry and maturation of pluripotency, similar to what was observed with MEFs.

Like in the case of MEF reprogramming, cell growth was slightly decreased at early period (d0 to d4), and drop in the cell growth at later stage (d8 to d18) was less prominent (Fig. 2F) in pAAm gel compared to TCPS. At the end of reprogramming, cell number under the pAAm gel condition was 2 times higher compared to the TCPS (Fig. 2F). This increase, combined with the higher number of bona fide hiPSCs (SSEA-4^+^TRA-1-60^+^), resulted in 10 times higher yield of hiPSC from the pAAm gel compared to the TCPS (Fig. 2G). These results demonstrated that, like MEF reprogramming, the pAAm gel condition accelerated human fibroblast reprogramming process by increasing the conversion of cells to prereprogrammed states during early stage and entry into a fully reprogrammed state during the final stage of reprogramming.

### Hydrogel produced iPSCs with characteristics different from TCPS-produced iPSCs

To determine how the derivation of miPSC on 102 kPa gels impacted gene expression during reprogramming, we subjected triplicate samples at each time point to RNA-seq and assessed total RNA expression in hydrogel and TCPS reprogrammed murine iPSCs following 5 passages on TCPS.

Bulk GSEA with DEGs at d17 between TCPS and pAAm miPSCs for various MIKKELSEN gene sets [20] in GSEA demonstrated that iPSC in pAAm up-regulated dedifferentiated state gene sets and down-regulated gene sets that are inactive in ESC and active in MEF (Fig. S6A; see the “Bioinformatics analysis” section for details) compared to the TCPS condition.

**Fig. 3.**
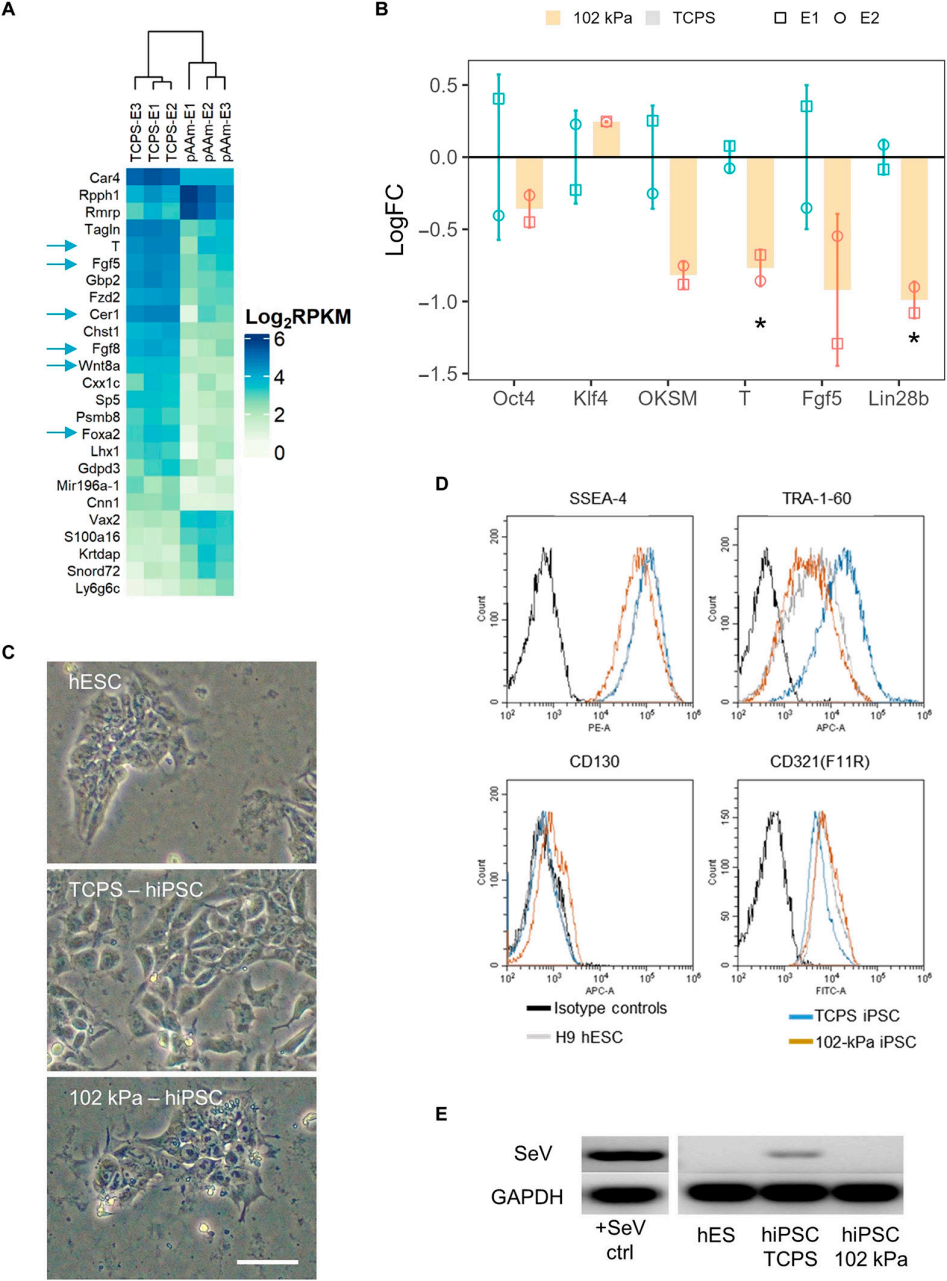
Characterization of miPSC (A and B) and hiPSC (C to E) produced on pAAm and TCPS. (A) Heatmap of most varied 25 DEGs that separated Oct4 GFP^+^ cell population at d17 in TCPS and pAAm. Arrows indicate the genes that are discussed in the result text. (B) Expression level of common PSC (Oct4 and Klf4) and primed markers (T, Fgf5, and Lin28b) and OKSM in miPSCs from pAAm and TCPS after subculturing them 5 times in TCPS (*n* = 2). Line at *y* = 0 represents mean expression in TCPS. Error bar represents means ± SD, and *P < 0.05 compared to TCPS. FC, fold change. (C) hiPSC morphology, (D) flow cytometry analyses of pluripotency-associated cell surface makers (that vary depending on hESC-primed status), and (E) residual Sendai virus (SeV) expression quantified by reverse transcription PCR and gel electrophoresis in hESC (H9) and hiPSC from TCPS and pAAm gel condition (*n* = 1). hiPSCs from both pAAm and TCPS condition were subcultured 8 times under TCPS condition before performing all the experiment. Scale bar, 50 μm. FITC, fluorescein isothiocyanate; GAPDH, glyceraldehyde-3-phosphate dehydrogenase.

At an individual gene expression level, a heatmap (Fig. 3A) of most varied DEGs at d17 showed that mouse Oct4 GFP^+^ cells under the gel condition down-regulated various markers that are highly expressed in primed mESC (Fgf5, T, Foxa2, Fgf8, Wnt8a, and Cer1) [34–36]. Notably, these markers were in the top 25 genes that varied most between TCPS and pAAm conditions, indicating that the primed status of miPSC was a significant factor discerning pAAm from TCPS. Moreover, miPSC on pAAm showed down-regulation of Sox17 and Lin28b and modest up-regulation of Klf4, Tbx3, and Tfcp2l1 (Figs. S6B and S7), which are up- and down-regulated in primed mESC, respectively [34–36].

Next, we investigated whether the difference observed in passage 0 iPSC could also be observed after expansion of miPSCs from pAAm and TCPS under TCPS conditions. Interestingly, even after subculturing 5 times on TCPS, qPCR analysis showed that miPSCs originally derived on the pAAm gel down-regulated primed mESC markers (T and Lin28b) and showed lower expression trends for OKSM transgene and Fgf5 compared to the TCPS-derived miPSCs (Fig. 3B). Overall, these observations indicated that pAAm-produced miPSCs were characteristically different (i.e., presumably less primed) compared to the miPSCs produced on TCPS. Next, we wished to investigate whether these observations were also translated in the case of hiPSCs.

After expanding hiPSCs 8 times from both pAAm and TCPS under TCPS conditions, hiPSCs from the gel condition were morphologically more like the H9 human ESCs (hESCs) when compared to the TCPS-derived cells (Fig. 3C) and exhibited a slower growth rate. Moreover, like the observed changes in expression pattern depending on the primed status of stem cells [37,38], hiPSCs derived from pAAm showed lower expression of SSEA-4 and TRA-1-60 and higher F11R and CD130 compared to TCPS-derived cells (Fig. 3D). We also observed that hiPSCs from pAAm almost lost all Sendai virus vector expression, whereas TCPS still had significant residual expression (Fig. 3E). Overall, these results suggested that, like miPSCs, pAAm-produced hiPSCs are characteristically different when compared to TCPS-produced hiPSCs and that the reprogramming process on hydrogel facilitated faster removal of exogenous factors used for reprogramming.

Last, we confirmed that embryoid bodies generated from both miPSC and hiPSC sourced from TCPS and 102 kPa pAAm gel differentiated into cell types that expressed genes representative of each of the 3 germ layers, and hiPSC lines from both substrates were karyotypically normal (Fig. S8A to C).

### Hydrogel modulates signaling and metabolic pathways that support faster reprogramming kinetics and distinct iPSC characteristics

**Fig. 4.**
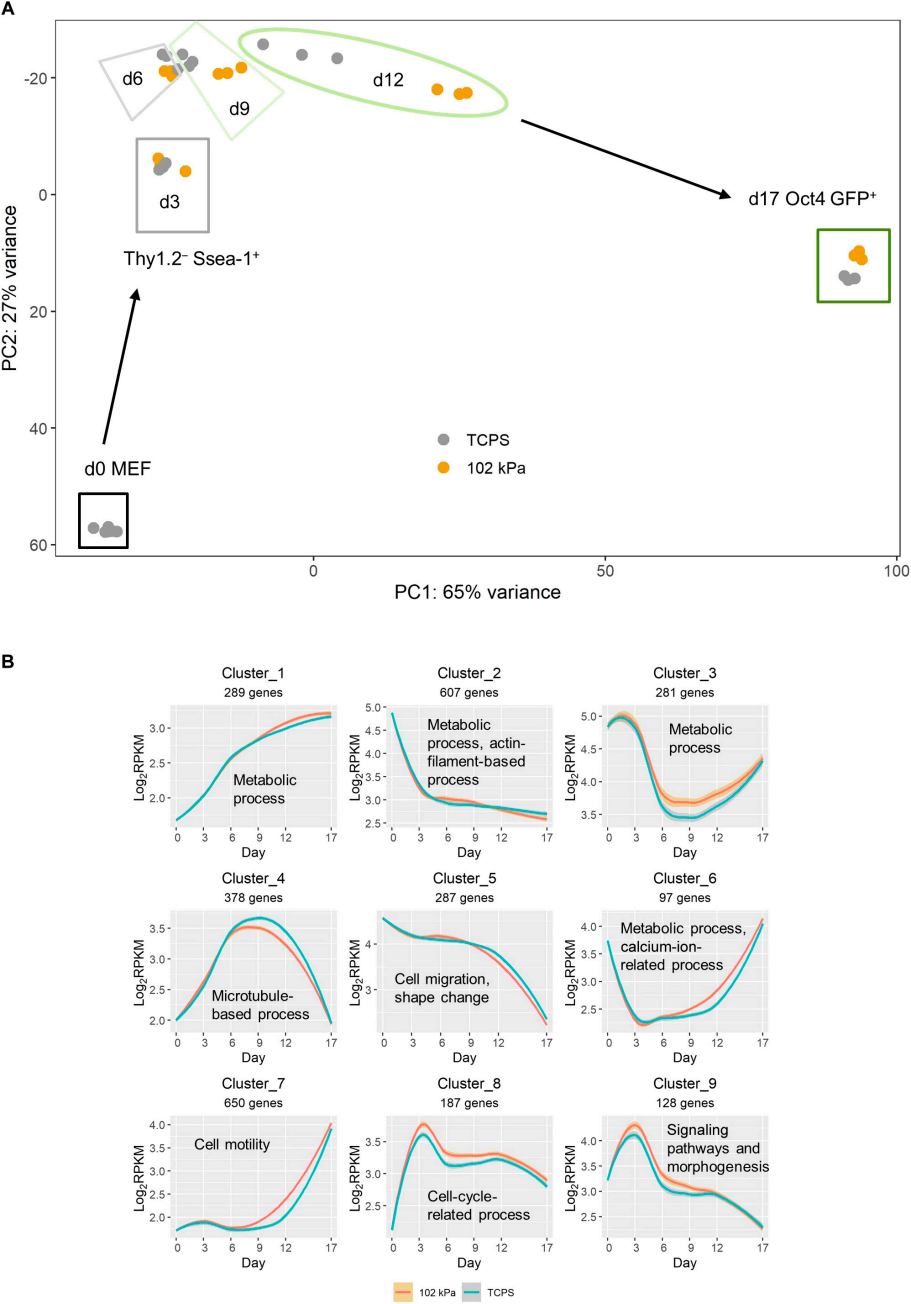
Characteristics of overall gene expression (quantified by RNA-seq) change during reprogramming under different substrate conditions. (A) PCA showing progress of different populations over time. (B) DEGs (FDR = 0.01) between pAAm and TCPS at different time points were clustered hierarchically and contrasted with the corresponding expression in TCPS. Band enclosing cluster or expression profile in (B) represents 95% confidence interval. Text label in each plot representative of significantly regulated pathways obtained in Panther GO-SLIM analysis with the corresponding genes in a cluster.

In a PCA (Fig. 4A) of RNA-seq data, d3 and d6 reprogramming intermediates (Thy1.2^-^Ssea-1^+^) from TCPS and pAAm gel clustered together, indicating similar kinetics during the early reprogramming period. However, intermediates in pAAm and TCPS at d9 and d12 did not cluster together, and a large separation was observed between them at d12. Moreover, compared to the TCPS, the separations between d9 and d12 clusters (Thy1.2^-^Ssea-1^+^ cells) and between d12 and d17 Oct4 GFP^+^ clusters were larger and smaller in case of pAAm gel, respectively, indicating faster progression of reprogramming intermediates to iPSC on the gel. These results also correlated well with the ability of pAAm gel to accelerate production of GFP^+^ iPSCs from an earlier time point compared to the TCPS (Fig. 1F). Finally, at d17, Oct4 GFP^+^ bona fide iPSC generated from TCPS and pAAm gel clustered together.

The PCA plot indicated that reprogramming on pAAm and TCPS followed a similar reprogramming route. To confirm this, we investigated overall expression profiles of genes that were clustered under key biological processes. Clustering of DEGs (with an FDR of 0.01) between TCPS and pAAm at any time point within d3 to d12 and then profiling their temporal expression showed that profiles (Fig. 4B) in TCPS and pAAm followed the same trend but that their expression levels differed at time periods (e. g. up-regulation of cluster 3 gene set from d6 to d12 in pAAm compared to TCPS). The close proximity of these profiles at d17 was in accordance with the observation made in the PCA analysis: closely clustered Oct4^+^ miPSC populations from TCPS and pAAm. Overall, these observations indicated that, rather than taking a different route through reprogramming, pAAm modulated cell reprogramming by more pronounced up- or down-regulation of key genes.

**Fig. 5.**
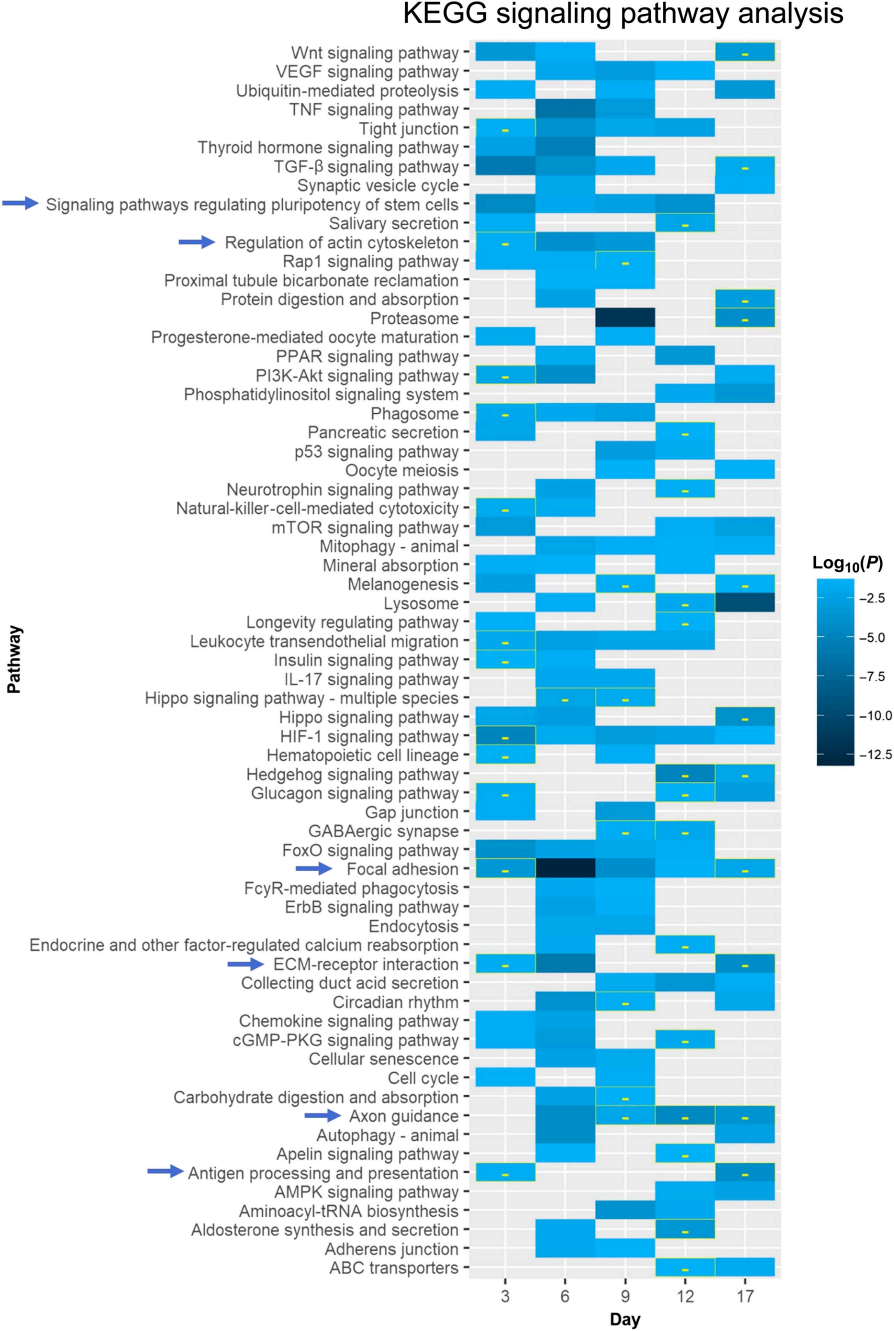
Up- and down-regulated KEGG signaling pathways in pAAm at different time points compared to TCPS. All tiles indicate up regulation except “-” symbol containing tiles. Arrows indicate the pathways that were discussed in the text. VEGF, vascular endothelial growth factor; TNF, tumor necrosis factor; TGF-β, transforming growth factor-β; PPAR, peroxisome proliferator–activated receptor; PI3K, phosphatidylinositol 3-kinase; mTOR, mammalian target of rapamycin; IL-17, interleukin-17; HIF-1, hypoxia-inducible factor 1; GABAergic, γ-aminobutyric acid-releasing; cGMP, guanosine 3′,5′-monophosphate; PKG, cGMP-dependent protein kinase; AMPK, adenosine 5′-monophosphate kinase; ABC, adenosine 5′-triphosphate-binding cassette.

Focal adhesion, ECM–receptor interaction, and regulation of actin cytoskeleton, all of which characterize cell engagement with the underlying substrate, were down-regulated at d3 but were up-regulated from d6 to d12, at d6 and from d6 to d9, respectively (Fig. 5). Pathways regulating pluripotency of stem cells were up-regulated under the pAAm condition from d3 to d12. At d17, iPSCs on pAAm down-regulated pathways related to cell adhesion (focal adhesion, ECM–receptor interaction, and cell adhesion molecules) as well as axon guidance and antigen processing and presentation pathways compared to TCPS (Fig. 5), which are reported to be down-regulated in mESC [39].

**Fig. 6.**
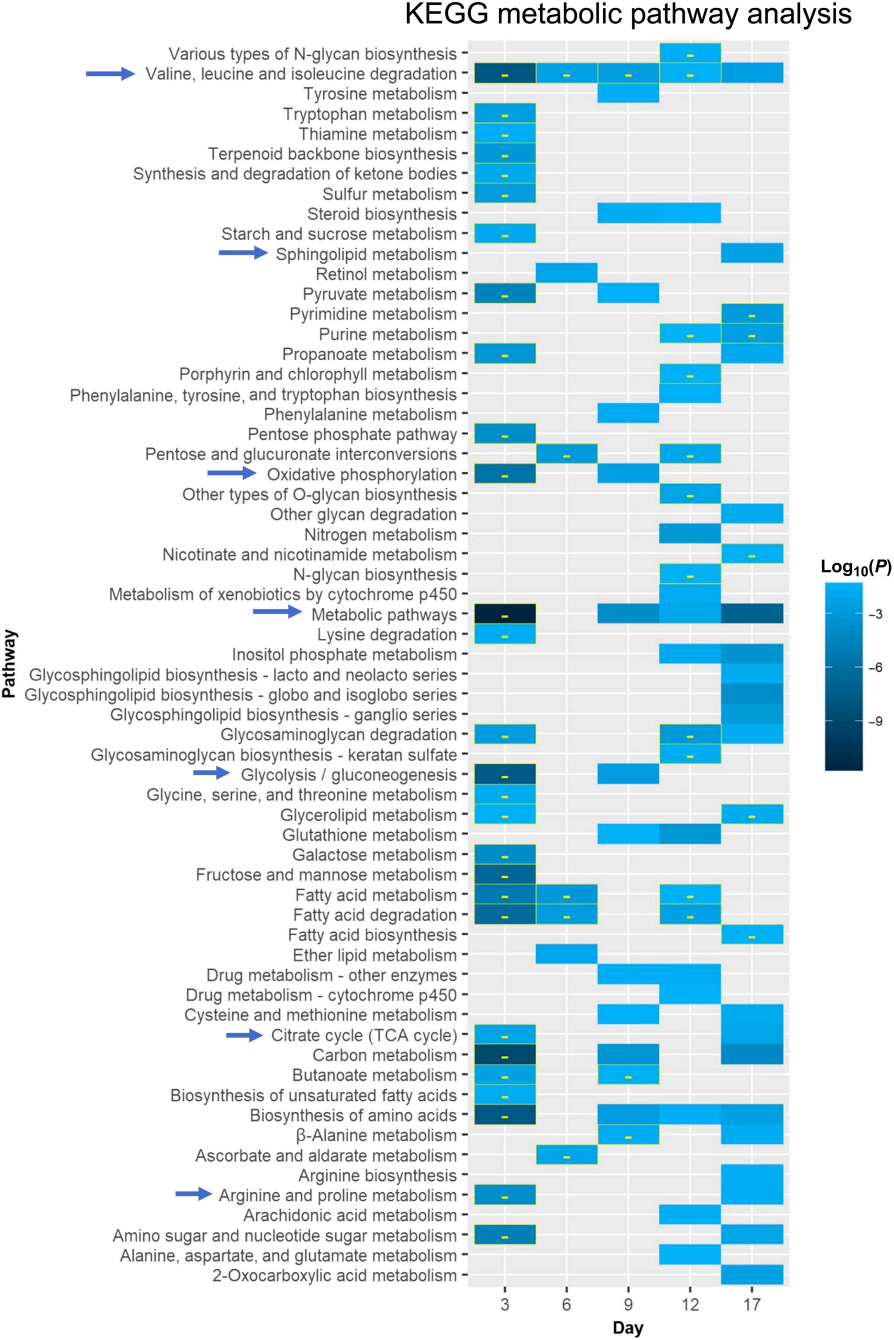
Up- and down-regulated KEGG metabolic pathways in pAAm at different time points compared to TCPS. All tiles indicate up regulation except “-” symbol containing tiles. Arrows indicate the pathways that were discussed in the text.

At d3, reprogramming cells in pAAm gel significantly down-regulated many metabolism pathways [which included glycolysis/gluconeogenesis, citrate cycle, and oxidative phosphorylation (OxPhos)] compared to TCPS (Fig. 6). After d3, cells on the pAAm hydrogel started to up-regulate various metabolic pathways, which were maximized at the end of reprogramming. Both glycolysis and OxPhos show rapid increase and decrease (forming a bell-shaped pulse) around d3 to d4 of reprogramming before their gradual increase (glycolysis) and decrease (OxPhos) over the remaining period of reprogramming [40]. These data and faster kinetics of reprogramming under the pAAm condition suggested that the down-regulation of metabolic pathways at d3 and up-regulation thereafter might have represented a faster shift of metabolism under the pAAm condition compared to TCPS.

Although OxPhos and glycolysis/gluconeogenesis were similar in iPSCs generated on the pAAm gel and TCPS on d17, sphingolipid, various amino acid metabolism, and tricarboxylic acid (TCA) cycle were up-regulated under the pAAm condition (Fig. 6). Up-regulation of the TCA cycle, along with down-regulation of Lin28b in less primed pAAm iPSC, correlated well with the known Lin28b effect on PSC metabolism and their transition from naïve to primed [35]. Overall, regulation of both signaling and metabolic pathways under the pAAm condition is consistent with its supportive role in facilitating iPSC production more efficiently compared to the TCPS condition.

### Phactr3 critically impacted the early-stage and end-stage outcomes of cell reprogramming

We next focused on identifying molecular targets that appear sensitive to substrate stiffness at the early stages of reprogramming for 2 reasons: Entry into a reprogramming prone state at the early stage is a bottleneck for the overall reprogramming process and efficiency, and cell crowding and cell-remodeled ECM become additional important factors at the later stages of reprogramming, potentially overshadowing other substrate-induced impacts.

By investigating DEGs at d3, we identified various molecular pathways, including Bmp, Fgf, and Gata TFs (Figs. S9A and S10; see the “Bioinformatics analysis” section for details), which are known to accelerate cell reprogramming [28,30,41]. In terms of the Bmp signaling pathway, the pAAm condition up-regulated various factors: Bmp2, Acvr1, Smads (4 and 9), and Ids (1 to 3) (Figs. S9 and S10). Hayashi and coworkers [41] reported increased cell reprogramming efficiency by Ids (1 to 3), in which up-regulation is mediated by Bmp/Acvr1 signaling. Bmp signaling contributes to established signaling pathways regulating pluripotency of stem cells and is crucial for MET and successful reprogramming under conventional TCPS culture conditions [41,42]. Choi et al. [9] suggested that improvement in cell reprogramming by short-term hydrogel exposure was similarly due to MET. Overall, these results on the 102 kPa substrate indicated that Bmp signaling played a crucial role in improving cell reprogramming under the pAAm gel condition at the early stages.

Further investigation of the protein–protein interaction network of DEGs at d3 showed that a relatively unknown protein, Phactr3, was up-regulated under the gel condition but was not connected with other genes (Fig. S9B), reflecting that very little is known about its involvement in biological processes, especially in cell reprogramming. Phactr3 belongs to a novel protein family containing 4 Phactr proteins (Phactr1 to Phactr 4) [43]. Besides the myocardin-related TF family, these are the only proteins containing a highly conserved RPEL domain that binds with G-actin, which acts as an actin monomer sensor in cells and modulates cell migration [44,45]. Phactr proteins also bind with protein phosphatases, which regulate metabolism, cell cycle, and muscle contractility [46,47]. Interestingly, Phactr3 is the only known Phactr associated with nuclear nonchromatin structure [45], therefore having the potential to influence the reprogramming process critically, as nuclear nonchromatin structures plays a core regulatory role in gene expression [48]. We thus next sought to validate the role of Phactr3 under standard reprogramming conditions, believing that it may connect the earliest changes in cytoskeleton and metabolism, influencing the reprogramming process under the hydrogel condition.

**Fig. 7.**
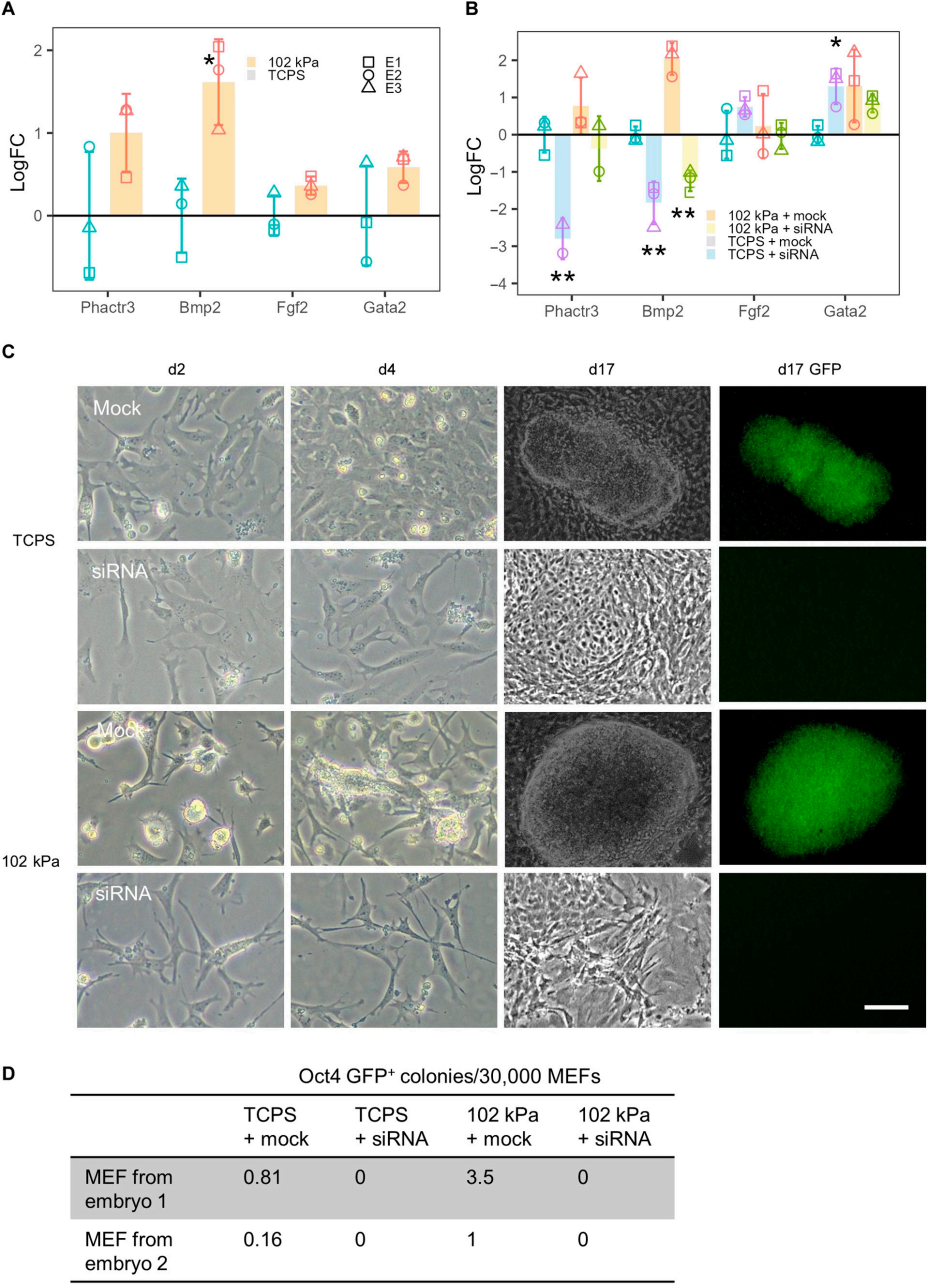
Regulation of cell reprogramming by Phactr3. (A) Phactr3 and other related gene expression (log fold change relative to TCPS) in MEF inoculated in TCPS and 102 kPa overnight. (B) Same genes expression in MEFs reprogrammed for 4 d with scrambled pool of siRNA (mock) or Phactr3 siRNA in TCPS and 102 kPa. E1, E2, and E3 represents MEFs from different embryos, and missing data point for any embryo represent not detected expression in qPCR. Line at *y* = 0 in (A) and (B) represents mean expression level in TCPS. Morphological observations in MEF reprogramming cultures at different time points (C) and (D) Oct4 GFP^+^ miPSC colony counts (mean from 2 wells or hydrogels on coverslips for each condition) at d17 in TCPS and 102 kPa with or without Phactr3 knockdown for 4 d. Data are represented as means ± SD (*n* = 3), and **P < 0.01 and *P < 0.05. Comparisons are TCPS versus pAAm for (A) and TCPS + mock versus TCPS + siRNA and 102 kPa + mock versus 102 kPa + siRNA for (B). Scale bar, 250 μm (for d2 and d4) or 100 μm (for d17 and d17 GFP). All images are in grayscale. miPSC colony counts are as number per 4.8 cm^2^.

qPCR analysis on MEF samples (inoculated on TCPS and 102 kPa for overnight) showed that Phactr3 expression was almost 2 times higher under the hydrogel condition compared to TCPS (Fig. 7A). Phactr3 expression in MEFs from different embryos showed high variability, but its up-regulation in the pAAm condition was consistent for MEFs from each embryo. Immunostaining of Phactr3 also confirmed qPCR data (Fig. S11A). These data confirmed that Phactr3 up-regulation is an early event and the hydrogel itself can induce its expression without Dox-inducible TFs. Interestingly, among the previously reported reprogramming facilitating factors (Bmp2, Fgf2, and Gata2) [28,30,41], only Bmp2 expression was significantly higher in MEFs incubated overnight on the pAAm substrate.

As the Bmp2 pathway is critical to inducing MET in reprogramming in the early stage, we predicted that if Phactr3 had a key influence over the Bmp2 pathway, supressing the expression of Phacrt3 would essentially supress the Bmp2 pathway, causing suppression of MET and iPSC colony formation. To substantiate our prediction, we suppressed Phactr3 expression in the early stage of reprogramming using siRNA. Application of siRNA against Phactr3 for 4 d during the reprogramming culture efficiently reduced its expression in the cells (Fig. 7B). For one MEF line, which showed the lowest Phacrt3 expression compared to other MEF lines, no expression of Phactr3 was detected under the siRNA condition. Upon Phactr3 knockdown with siRNA during a reprogramming culture for 4 d, Bmp2 expression was significantly down-regulated (Fig. 7B). Under Phactr3-knockdown reprogramming conditions, cells remained fibroblastic and failed to morph into epithelial-like cells and form clusters, a well-known characteristic change associated with MET at the early stage of reprogramming (Fig. 7C). Finally, suppression of Phactr3 for the first 4 d of reprogramming completely abolished the formation Oct4 GFP^+^ colonies under both TCPS and the 102 kPa gel conditions (Fig. 7D), indicating that Phactr3 pays a crucial role in the early stages of reprogramming. These results confirmed that Phactr3 up-regulation was one of the earliest events triggered by the hydrogel condition, and this up-regulation appeared to drive Bmp2 up-regulation, which ultimately lead to more efficient MET under the hydrogel condition, resulting in increased iPSC colony formation compared to TCPS.

## Discussion

In this study, we reported that the pAAm gel had a pronounced effect on both the early and later stages of reprogramming (Figs. 1 and 2), whereas other studies thus far have only reported biomaterial-based modulation of the early stage [9–12].

Downing and coworkers [10] reported improvement of miPSC or hiPSC colony production by 4 times and 2 times, respectively, on 10 μm microgroove-patterned polydimethylsiloxane surface compared to flat polydimethylsiloxane surface. Choi and coworkers [9] transduced mouse fibroblasts with reprogramming TFs at d1 on TCPS, plated them at d2 on the hydrogels of different stiffness (0.1 to 20 kPa) for 1 d and then replated the cells back onto TCPS for the remainder of the reprogramming period (another 18 d). They reported 2 times improvement in the reprogramming prone population (i.e., Thy1.2^−^/Ssea-1^+^) and 2 times more miPSC colony production after the 1 d of exposure of cells to the 0.1-kPa condition, compared to other stiffnesses. Caiazzo and coworkers [11] reported an improvement in reprogramming with very soft (0.6 kPa) 3D PEG-based hydrogels, which achieved 2.5-fold (at d16) and 2-fold (at 6-weeks) more miPSCs and hiPSC colonies, respectively. Kim and coworkers [12] only reported improvement in miPSC production using methacrylated hyaluronic acid hydrogel with a stiffness of 0.1 kPa. These studies highlighted the substrate-mediated improvement of MET during the early stage of reprogramming for the overall improvement of the reprogramming outcome, i.e., number of iPSC colonies.

In contrast to previous studies, we performed reprogramming continuously on 2D hydrogel substrates without the need for replating and observed 2 times more Thy1.2^−^/Ssea-1^+^ murine cells at d3 and 4 times more Oct4 GFP^+^ miPSC colonies at the end of reprogramming (Fig. 1). In the case of human fibroblasts reprogramming, we observed a pronounced impact in the later stages of reprogramming compared to the early stages under the hydrogel condition, resulting in 2-fold more hiPSC colonies and a 10 times greater yield of hiPSCs compared to TCPS in under 3 weeks (Fig. 2). We also observed that both miPSC and hiPSC from the pAAm condition had lower expression of primed ESC-markers and exogenous TFs used for reprogramming (i.e., OKSM or Sendai vector expression) compared to the TCPS condition (Fig. 3). 3D cell culture systems or patterned microgrooved substrates used for reprogramming in previous studies [10–12] are still challenging to be adopted in laboratories for routine production of iPSCs, which itself is a sensitive process. Overall, our study demonstrated 2D hydrogel-based improvement of both early and late phases of reprogramming and established an optimized pAAm hydrogel substrate (that can be easily made with a very low cost) platform as a robust 2D surface for accelerated manufacturing of quality iPSCs.

Another notable observation is that the hydrogel determined to be optimum for reprogramming in this study is much stiffer than the hydrogels reported in previous studies (<1 kPa) [9,11,12]. In contrast to the results reported by Choi and coworkers (lower percentage of Ssea-1^+^ population in stiffness of >4 kPa than <1 kPa), we observed that hydrogels of all stiffness (1 kPa to 1.3 MPa) produced more Ssea-1^+^ population than TCPS at d3 (Fig. S4B). Notably, Choi and coworkers analyzed for the percentage of Ssea-1^+^ population after only 1 d of exposure of the already transduced mouse fibroblasts (on TCPS) on the hydrogel substates, whereas we analyzed at d3 after transduction on the same substrates. After 1 d of exposure to their hydrogels, Choi and coworkers [9] replated the cells onto TCPS for the remaining reprogramming period. However, we managed to perform the reprogramming on the hydrogel from start to finish, representing a very different, simpler approach. Our results indicate that, if enough time is given, stiffer hydrogels (in the range of kilopascal to megapascal) have an equal potential to form increased numbers of reprogramming-prone populations at the early stage of reprogramming. We observed that cell growth rate was generally lower on hydrogels and, moreover, that hydrogels below ≤16 kPa could not recover growth rates at later stages of reprogramming (Fig. S4A). Choi and coworkers [9] investigated pAAm hydrogels of stiffness of 0.1 to 20 kPa, which fall in the range we found not to be supportive of cell growth, potentially providing reasoning for the need to replate the cells on TCPS for the remainder of the reprogramming period in their experiments. In contrast, we were able to identify an optimum hydrogel of a higher stiffness (102 kPa) that improved both early and late stages of reprogramming, without impacting final cell growth outcomes. It is worth noting that in previous works investigating 3D hydrogels for reprogramming (of stiffnesses of <1 kPa), a high number of cells (1 to 2 M/ml) were required to optimize the reprogramming outcome [11,12] as very soft hydrogels impart growth limitations at low to moderate cell densities [11]. Improvements in reprogramming in our pAAm hydrogels compared to reports on these 3D hydrogels were found to be slightly higher on the same comparators, being 4-fold versus 2.5-fold more Oct4 GFP^+^ miPSC colonies generated (hiPSC colonies were measured using different markers). The 2D pAAm hydrogel format offers significant advantages over 3D alternatives, not only in being a low-cost, easily made, and scalable platform in itself but also for further optimization with the inclusion of other biophysical cues (e.g., microtopography and light-induced patterned region) and small molecules.

In this study, we have identified a novel mechanosensitive molecule, Phactr3, which was up-regulated under the pAAm gel condition at the earliest time point, without any influence from the exogenous TF expression, and confirmed it to be crucial for Bmp2-mediated MET and reprogramming (Fig. 7). In addition to Phactr3 up-regulation, various genes related to metabolism (Hdac11, Pygl, Ppp1ca, and Ppp2ca) were up-regulated in MEFs exposed to the pAAm gel overnight (Fig. S11B). As Phactr proteins bind with protein phosphatases, which regulate metabolism, cell cycle, and muscle contractility [43–47], it is highly plausible that Phactr3 is also associated with the observed significant metabolic changes under the hydrogel condition at the early stage of reprogramming. Understanding the exact role of Phactr3 (and other Phactrs) on metabolism during reprogramming requires further investigation of the early-stage reprogramming process at shorter time spans (i.e., less than 3 d), as metabolism changes rapidly in these stages [40]. Future studies exploring the impacts of topography, 2D topography-patterned hydrogel or 3D gel conditions on Phactr3 expression, and its impact on reprogramming and other cellular process outcomes would also be useful.

The large RNA-seq dataset generated in the study contains 6 time point samples (d0 MEF, reprogramming intermediates from d3 and d12, and Oct4 GFP^+^ iPSCs at d17) from 2 substrate conditions. In this study, we explored only some of the possible molecular targets at d3, leaving potential opportunity to explore more (e.g., role of noncanonical Wnt signaling, as Wnt7a was highly up-regulated and its association with Phactr3) and other time points (e.g., from d6, as from this point acceleration of reprogramming started in the pAAm gel condition). Although we observed large improvements in the later stage of reprogramming, it is not known as to whether the improvement is independent of the early stage of reprogramming. Further, the distinct impact of the early- and late-stage improvements on iPSCs characteristics, which was different between the gel and TCPS conditions, is not known. Future studies guided by the large RNA-seq dataset will pave the way to answering these questions and potentially lead to the establishment of a robust reprogramming culture substrate and methodology for producing quality iPSCs in a more cost-effective manner than current gold-standard practices for regenerative medicine applications.

By using an optimized hydrogel substrate, we confirmed the notion by Tapia and Schöler [2] that faster kinetics and efficient reprogramming impacts the quality or characteristics of iPSCs. Even after the bulk passaging (more than 5 times) of miPSC or hiPSC on the TCPS, characteristics of iPSC produced under the pAAm hydrogel condition was found to be different compared to the TCPS-produced iPSCs (Fig. 3), presumably indicating different epigenetic status. We also observed that both miPSC and hiPSC from the pAAm condition had less exogenous expressions of the TFs used for reprogramming (i.e., OKSM or Sendai vector expression) compared to the TCPS condition. Higher growth rates of cells at the later period of reprogramming (Figs. 1D and 2E) under the pAAm gel condition might be a reason for the accelerated loss of transgene expression. These data suggest that besides the observed significant influences over the “production” outcomes of the reprogramming process, the stiffness of a hydrogel may also have greater influence over genetic change/insults that occur over iPSC culture periods [2,49]. In this study, we did not assess the quality of iPSCs in a broader scope (e.g., immunogenic, genomic abnormalities, or epigenetic status) or long-term propagation of iPSCs on the respective surfaces (i.e., hydrogel or TCPS) they are produced from. Therefore, studies that further compare the quality of iPSCs produced from pAAm and TCPS from a broader perspective would be important to pursue, as iPSC quality strongly determines differentiation efficiency and quality of the differentiated cells [49].

## Conclusion

In this study, by screening a series of 2D pAAm hydrogels of varying stiffness, we found an optimum pAAm hydrogel condition (of stiffness of 102 kPa) that significantly boosted both miPSC and hiPSC production from fibroblasts compared to gold-standard TCPS-based protocols. This methodology permitted continuous reprogramming of fibroblasts on the hydrogel substrate, without the need for intermediate cell replating, even using Sendai virus methods for hiPSC generation. Using both experimental and time-course RNA-seq datasets, we demonstrated that the 2D hydrogel condition accelerated the reprogramming processes and modulated both early- and late-stage reprogramming, ultimately producing more iPSCs (10-fold) over the same time period (18 d) that had distinct characteristics compared to those produced using conventional TCPS-based culture practices. Furthermore, we identified a novel mechanosensitive protein, Phactr3, which critically impacted early-stage reprogramming and subsequently the end outcome of the reprogramming in both hydrogel-based and standard TCPS culture methods. These results indicate that the optimized 2D hydrogel platform discovered herein has significant potential as a robust platform for producing quality iPSCs more effectively. Future studies, supported by the large reprogramming time-course RNA-seq dataset made available by this study, focusing on the detailed assessment (e.g., immunogenic and epigenetic) of hydrogel-based modulation of iPSC quality, the impact of hydrogel surface modifications (e.g., photo-induced patterning and topography) on reprogramming outcomes, and the effect of the identified novel molecular target Phactr3, will likely produce further insight and maturation of hydrogel-based methodologies for cell reprogramming.

## Data Availability

All data generated or analyzed during this study are either included in this published article (and its Supplementary Materials) or are available from the corresponding author on reasonable request.
