# Supplementary material for: Superior Induced Pluripotent Stem Cell Generation through Phactr3-Driven Mechanomodulation of Both Early and Late Phases of Cell Reprogramming
Source: Biomater Res. 2024 May 21;28:0025. doi: 10.34133/bmr.0025 (PMC11106629; doi:10.34133/bmr.0025)
Supplement: Supplementary 1 — Figs. S1 to S11 [file bmr.0025.f1.zip › Manuscript_Chowdhury et al_supp_fig_legends.docx]

**Supplementary figure legends**

**Figure S1.** Rheological characterisation of polyacrylamide hydrogel (pAAm). (**a**) Storage (*G’*) and loss (*G’’*) modulus, measured by oscillatory rheometer, for representative pAAm gels prepared by varying acrylamide and bis-acrylamide amount in 29:1 ratio. Significantly lower value *G”* indicated mostly elastic behaviour of these gels. (**b**) Young’s Modulus (*E*) of pAAm gels calculated by using shear modulus (*G*) and Poisson’s ratio (ν = 0.457, as described in method section) (n = 2 or 3). (c) 102kPa hydrogel stiffness measured by Atomic force microscopy (AFM). Error bar represents Mean ± SD.

**Figure S2.** MEF reprogramming on pAAm gels of various stiffness (conjugated with Sulfo-SANPAH for ECM immobilization). (**a-1 – a-4**) Number of Oct4 GFP+ miPSC colonies count under microscopic observation at day 17 on TCPS and pAAm gel of various stiffness. (**a-1 – a-3**) During the initial screening, hydrogel of different stiffnesses were screened in batches at different times by running TCPS control in parallel as the objective was to find hydrogel stiffness that provide better reprogramming outcome than TCPS. Later on, the integrity of the batch experiments was confirmed by running a small experiment of which the outcome correlated well with batch experiments (**a-1** and **a-2**). MEFs from two or three embryos (E1, E2 and E3; n = 3) were used for reprogramming. Each data point reflects mean of two or three technical replicates (i.e., two or three wells or hydrogels on coverslips) for each MEF line. Error bars represent SEM and “*” represents *p* < 0.05 compared to TCPS. All comparisons were made by using TCPS as control. (**b**) Bright field and corresponding GFP channel showing miPSC colonies adhered on the surface of the 102 kPa stiff hydrogel (left) and with the detached cell layer (right) over the same stiff hydrogel. Scale bar represents 500 µm.

**Figure S3.** Characterisation of ECM immobilization on pAAm gel using Sulfo-SANPAH (SS) or L-DOPA (LD). (**a**) Images showing immunostaining of Col1A that was deposited on TCPS and pAAm (E = 102 kPa) treated with SS and LD (scale bar: 125 µm). (**b**) Intensity of the images quantified by ImageJ (n = 2 or 3). **(c)** representative images showing Coomassie blue staining for detecting immobilized Gelatin on hydrogel and **(d)** quantification (blank subtracted average gray scale value of LD+0.2% Gelatin) of the staining by image analysis in ImageJ showing the amount of Gelatin deposition on hydrogel of various stiffness. Representative images showing MEF morphology on different substrates after overnight incubation (e; scale bar: 250 µm). Cells were stained with Calcein AM or Dil stain. Cell area (**f**), Cell number per field after overnight incubation (**g**), and relative cell number quantified by presto blue assay at day3 (**h**). For (**e**) to (**h**), MEFs from two or three different embryos were tested. Data is represented as mean ± SEM and “****”, “***”, and “**” represents *p* < 0.0001, *p* < 0.001 and *p* < 0.01 compared to TCPS, respectively.

**Figure S4.** (**a**) Cell growth decrease (quantified by cell number) at early period (Day 3) of reprogramming but became similar to the TCPS at the end of reprogramming (Day 17) on pAAm gels for E ≥ 102 kPa. Soft substrate (*E* = 16 kPa) was not able to recover the initial drop-in growth rate at the end of reprogramming. (**b**) Percentage of cells undergoing reprogramming (Thy1.2- SSEA1+ cells) at Day 3 on TCPS and pAAm gel of various stiffness. Data from different batches of experiment is presented together. MEF from three embryos (E1, E2 and E3; n = 3) were used for reprogramming. Each data point reflects mean of two technical replicates for each MEF line. Error bars represent SEM and “***”, “**” and “*” represents *p* < 0.001, *p* < 0.01 and *p* < 0.05 compared to TCPS, respectively.

**Figure S5.** **(a)** hiPSC was stained (as described in Methods) for Alkaline Phosphatase (ALP). Images of ALP+ (red) hiPSC colonies at day 18 in TCPS (first two columns) and 102 kPa (last two columns). Each well of TCPS represents a well of 12-well plate and for hydrogel each represents a 22 mm x 22 mm coverslip. **(b)** Viability (by PrestoBlue assay) of hDFn (inoculated at different densities) on day4 after inducing them with Sendai virus for reprogramming. Data is for two hDFn cell lines and represented as mean ± SEM.

**Figure S6.** **(a)** Up or Down regulated Mikkelsen gene sets in MSigDB, which are related cell reprogramming to pluripotency. **(b)** Volcano plot of DEGs at day 17 between 102 kPa and TCPS (**b**). Horizontal and vertical dotted lines represent p cut-off value (0.01) logFC cut-off value (1), respectively.

**Figure S7.** Expression profile of various genes that were mentioned in the main text. Data represents mean ± SD.

**Figure S8**. (**a**) Karyotype of hiPSC generated in TCPS and pAAm. (**b**) Expression level of PSC markers (Oct4/OCT4 and Nanog/NANOG) and differentiation makers (remaining ones) in **(b)** mEB (n = 2, relative to TCPS) and **(c)** hEB (n = 1, relative to expression in hEB formed with H9 hESCs). Line at y=0 represents mean expression in TCPS. mEBs or hEBs were formed from miPSC or hiPSC derived in TCPS and pAAm and differentiated for 8-days in 20% Knockout Serum containing medium. Data is represented as mean ± SD.

**Figure S9.** (**a**) Volcano plot of DEGs at day 3 between 102 kPa and TCPS. Horizontal and vertical dotted lines represent *p* cut-off value (0.01) logFC cut-off value (1), respectively. (**b**) Network view of lowly connected (relationship retrieved from STRING database) DEGs (between pAAm and TCPS at day3) in Cytoscape. DEGs (FDR = 0.01) were grouped together according to their biological function or location in a cell as described by the corresponding GO terms (bold text). Colour indicates relative log-fold change, red is higher, and blue is lower.

**Figure S10.** Expression profile of various genes that were mentioned in the main text. Data represents mean ± SD.

**Figure S11.** (**a**) Comparison of Phactr3 expression between 102 kPa and TCPS by immunostaining after overnight incubation. (**b**) Metabolism related gene expression (Log fold change (LogFC) relative to TCPS) in MEF inoculated in TCPS and 102 kPa overnight. Line at y =0 represents mean expression in TCPS. Data is represented as mean ± SD (n = 3) and “***”, “**” and “*” represents *p* < 0.001, *p* < 0.01 and *p* < 0.05 compared to TCPS, respectively. Scale bar represents 50 µm.
