# Supplementary figures and images for: Superior Induced Pluripotent Stem Cell Generation through Phactr3-Driven Mechanomodulation of Both Early and Late Phases of Cell Reprogramming
Source: Biomater Res. 2024 May 21;28:0025. doi: 10.34133/bmr.0025 (PMC11106629; doi:10.34133/bmr.0025)

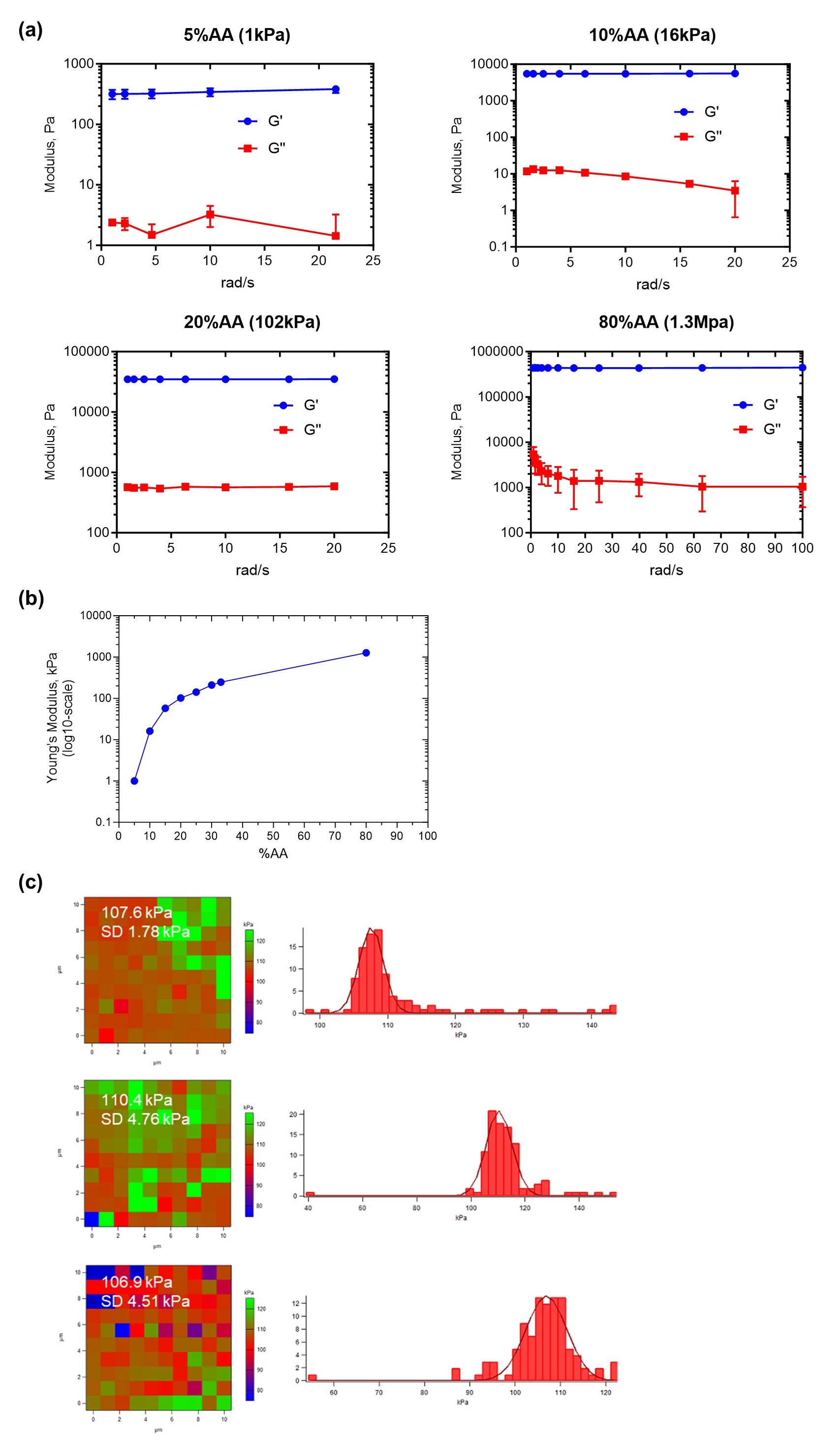

Supplement: Supplementary 1 — Figs. S1 to S11 [file bmr.0025.f1.zip › SuppFig1.jpg]

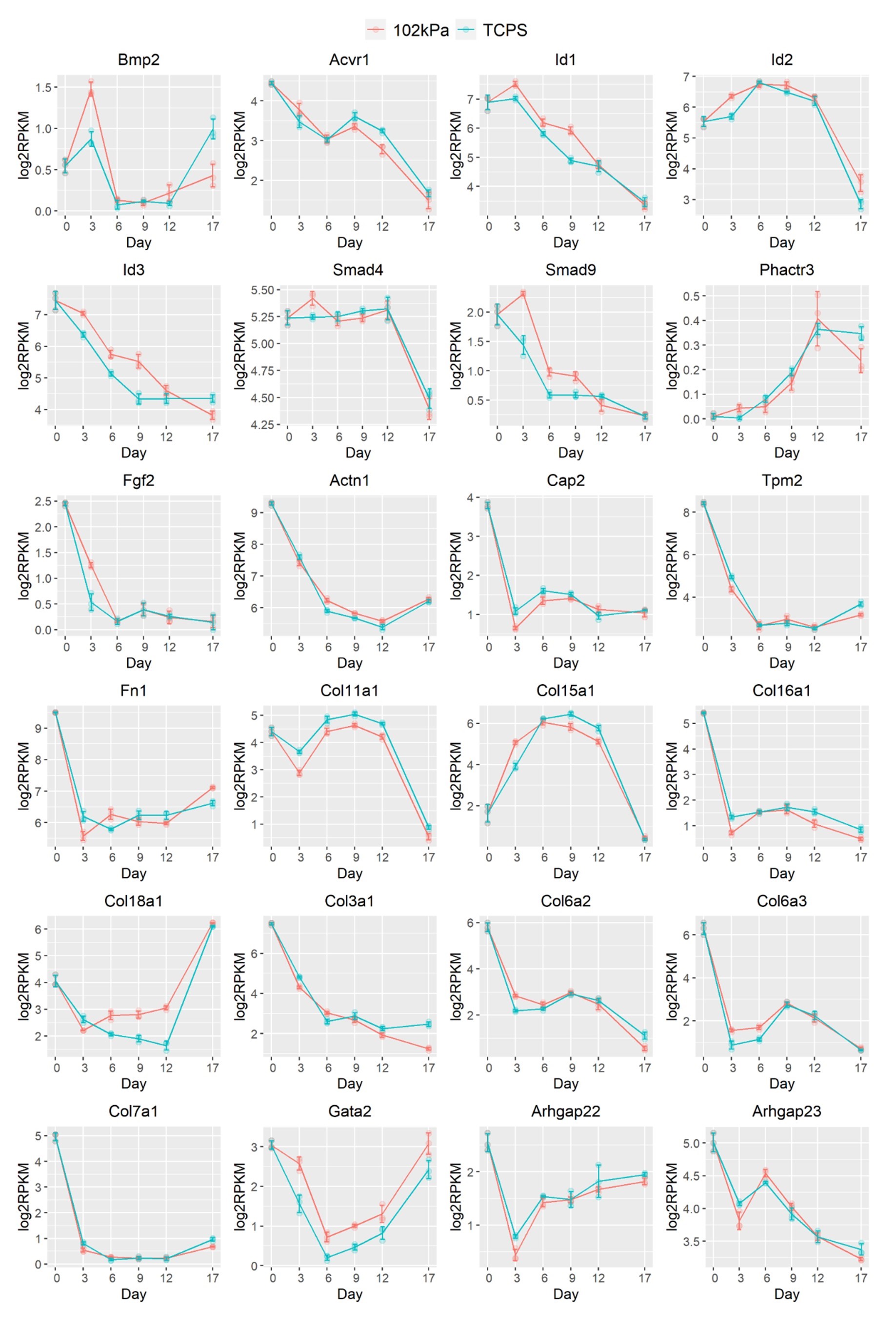

Supplement: Supplementary 1 — Figs. S1 to S11 [file bmr.0025.f1.zip › SuppFig10.jpg]

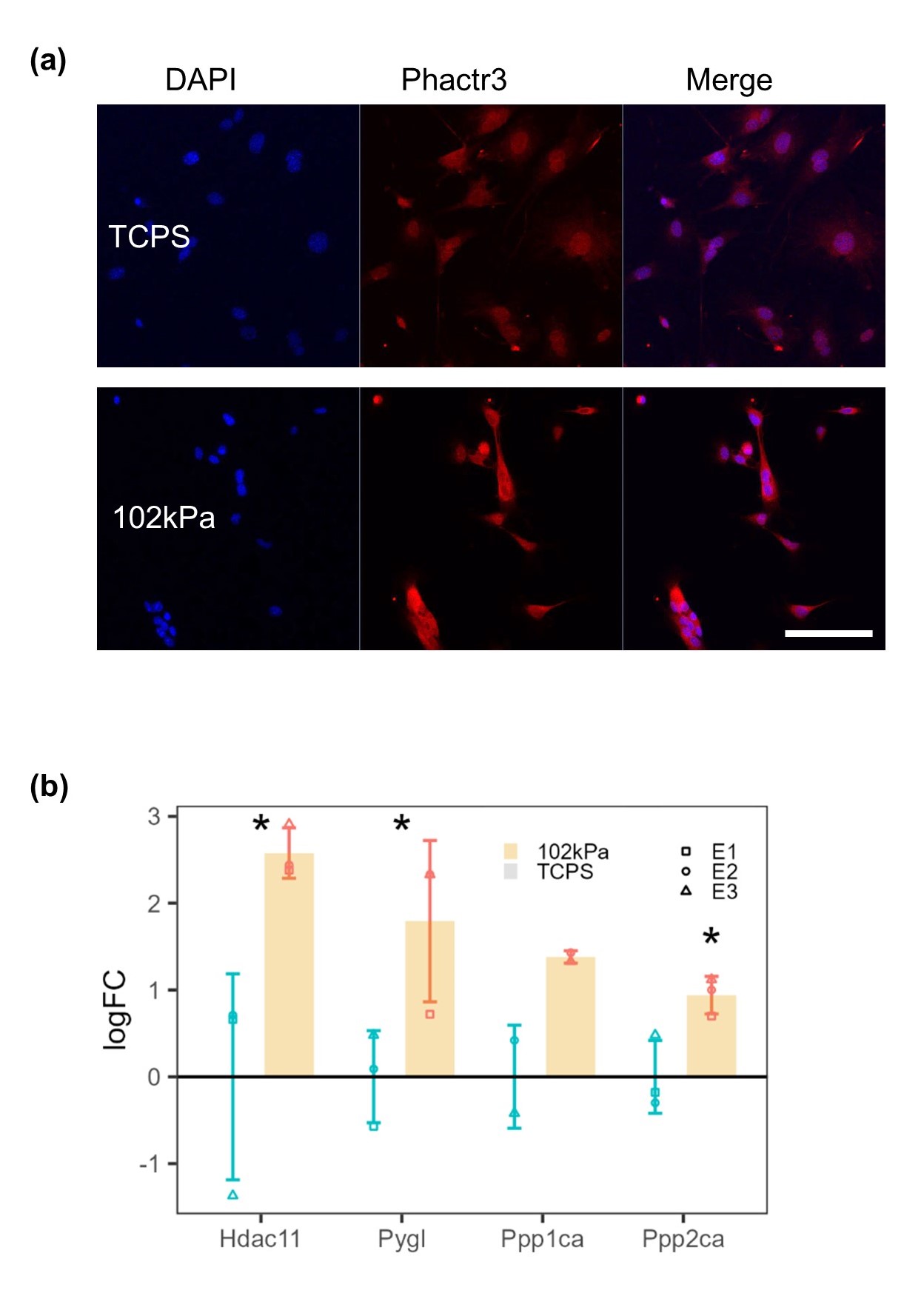

Supplement: Supplementary 1 — Figs. S1 to S11 [file bmr.0025.f1.zip › SuppFig11.jpg]

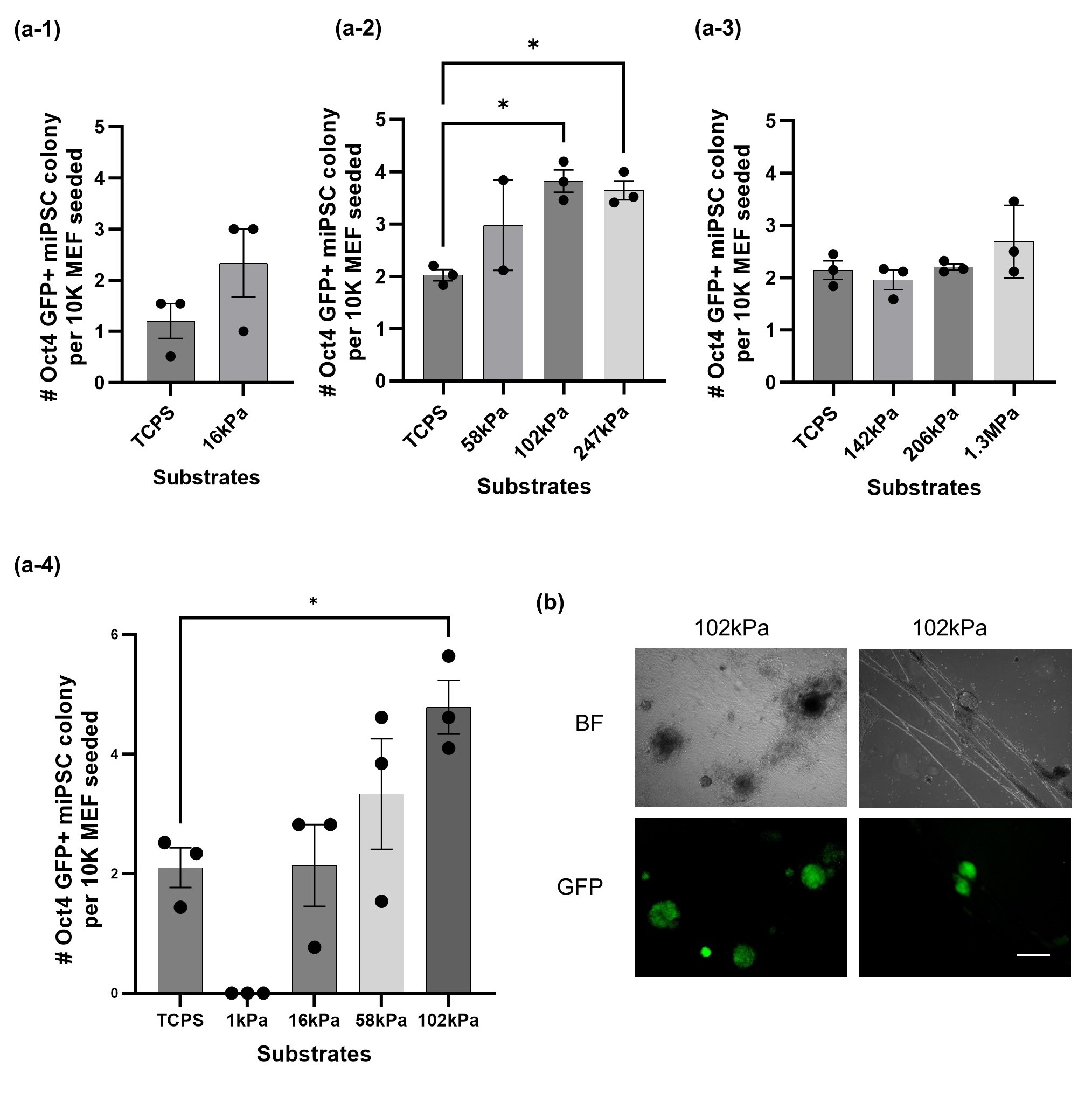

Supplement: Supplementary 1 — Figs. S1 to S11 [file bmr.0025.f1.zip › SuppFig2.jpg]

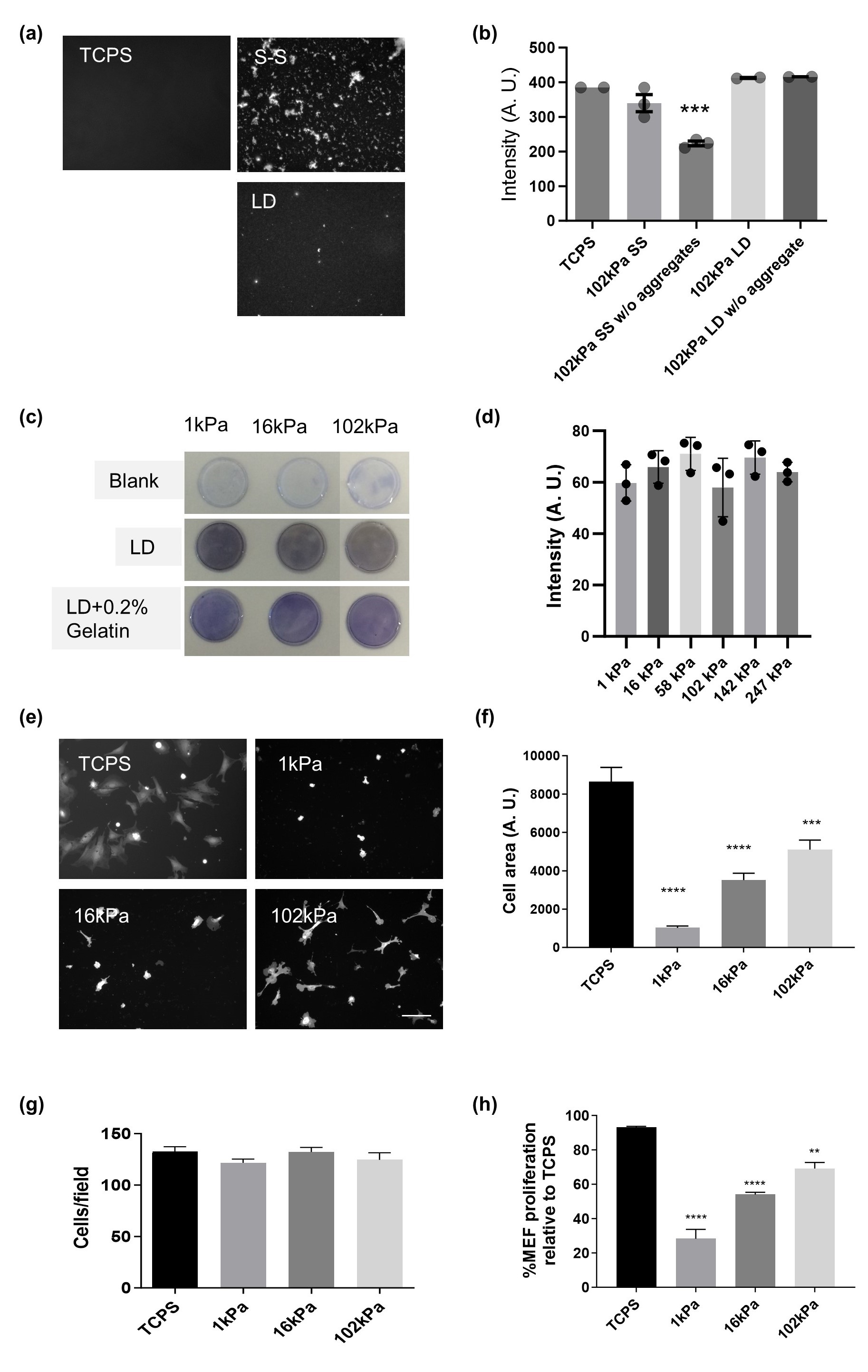

Supplement: Supplementary 1 — Figs. S1 to S11 [file bmr.0025.f1.zip › SuppFig3.jpg]

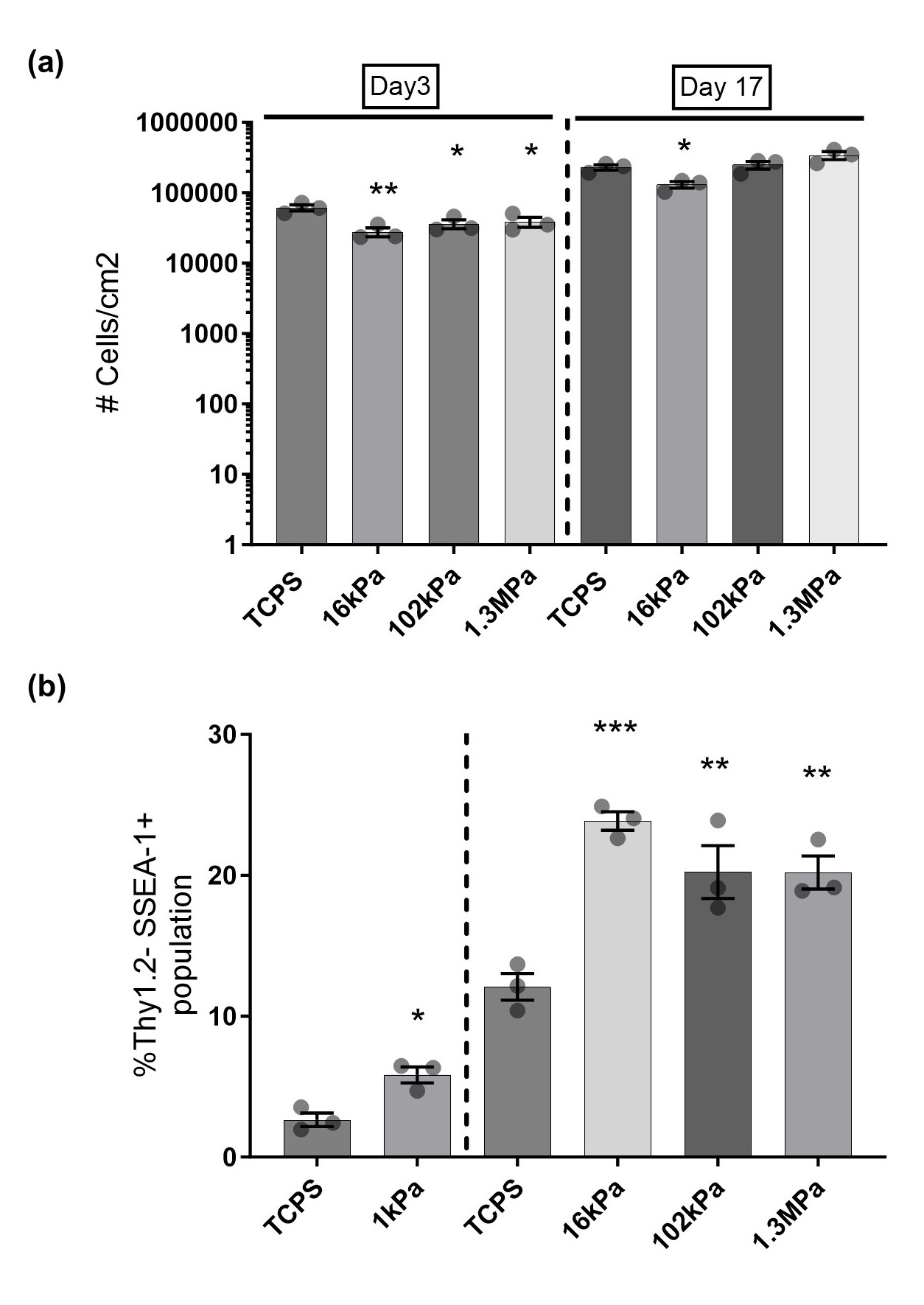

Supplement: Supplementary 1 — Figs. S1 to S11 [file bmr.0025.f1.zip › SuppFig4.jpg]

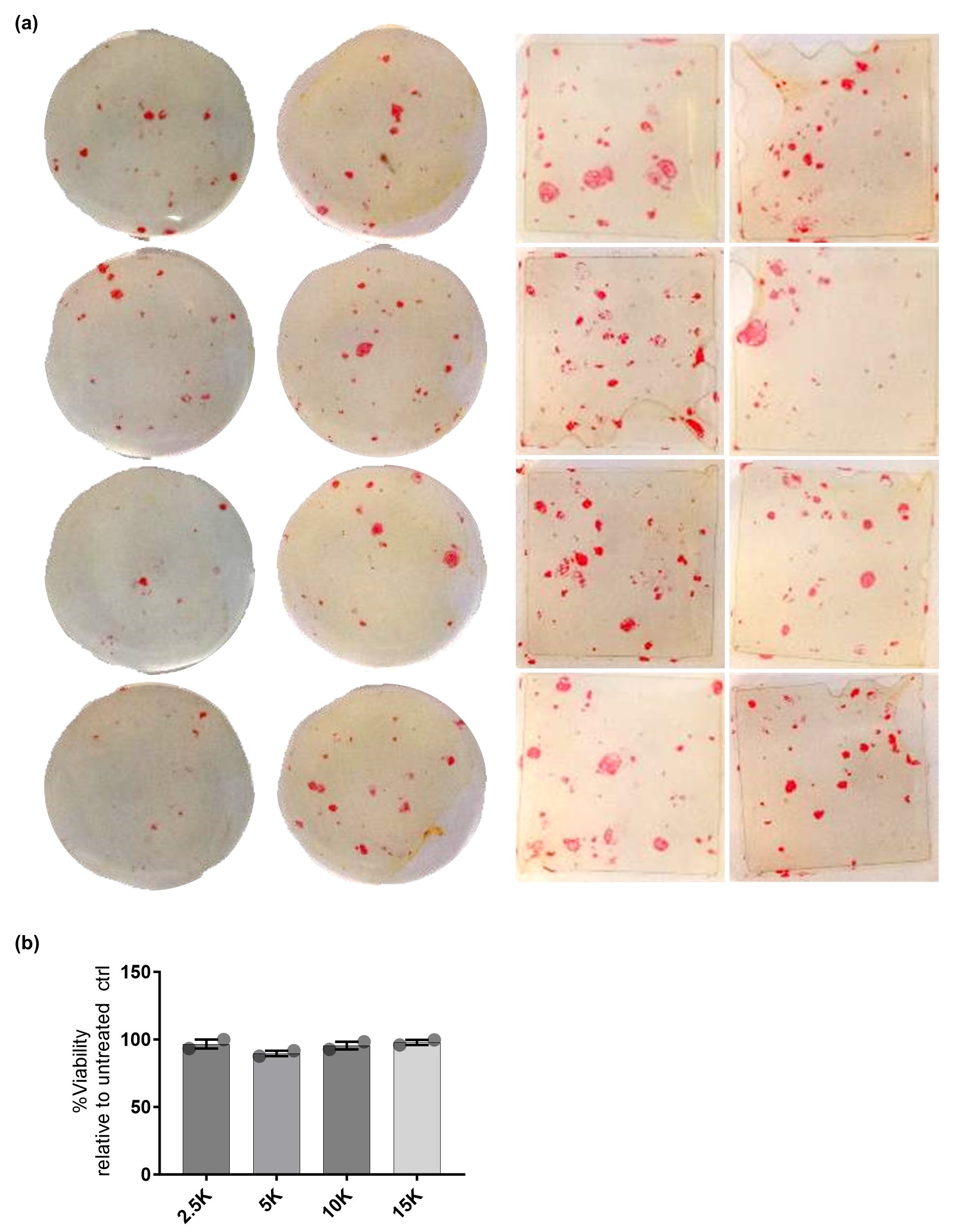

Supplement: Supplementary 1 — Figs. S1 to S11 [file bmr.0025.f1.zip › SuppFig5.jpg]

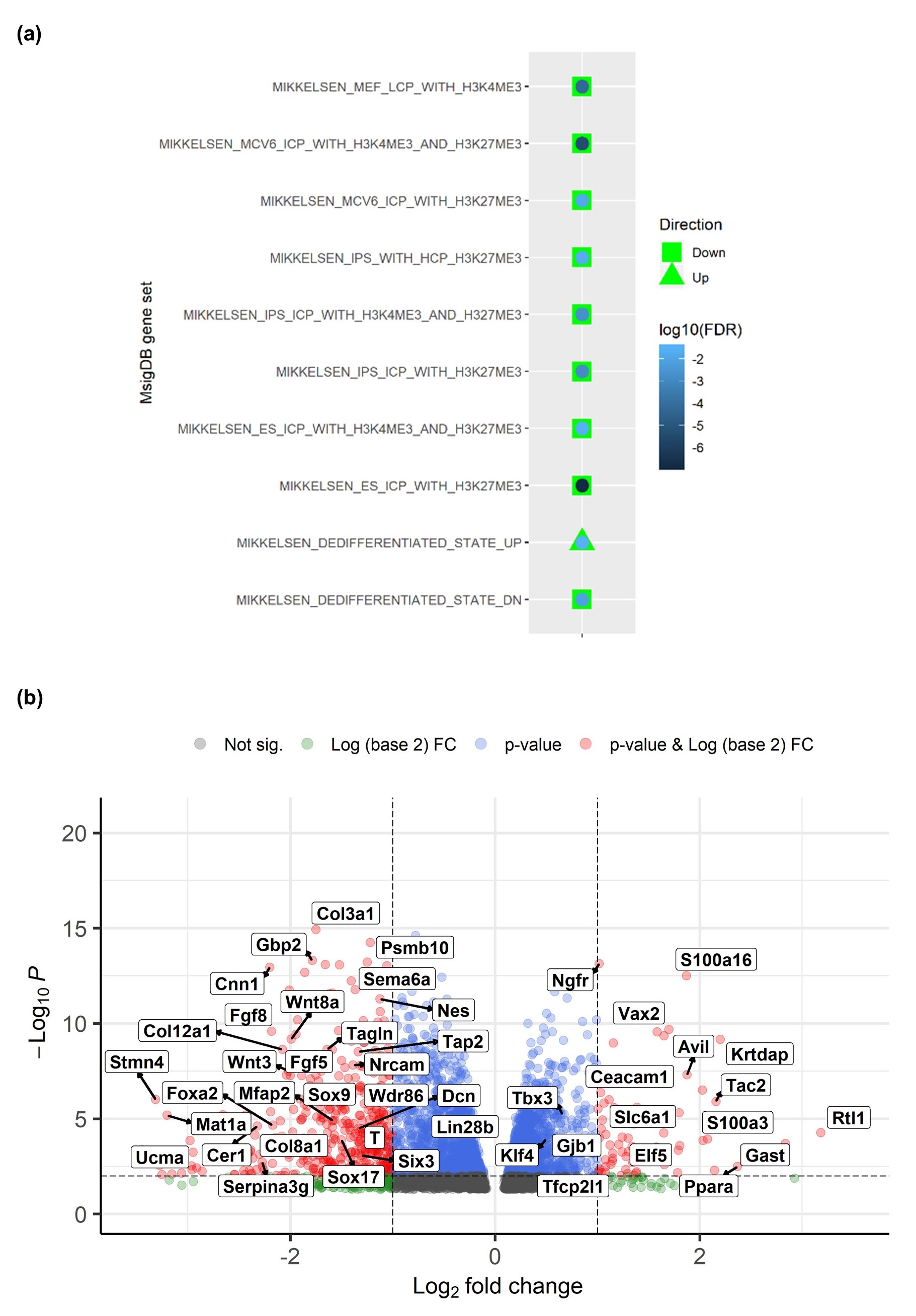

Supplement: Supplementary 1 — Figs. S1 to S11 [file bmr.0025.f1.zip › SuppFig6.jpg]

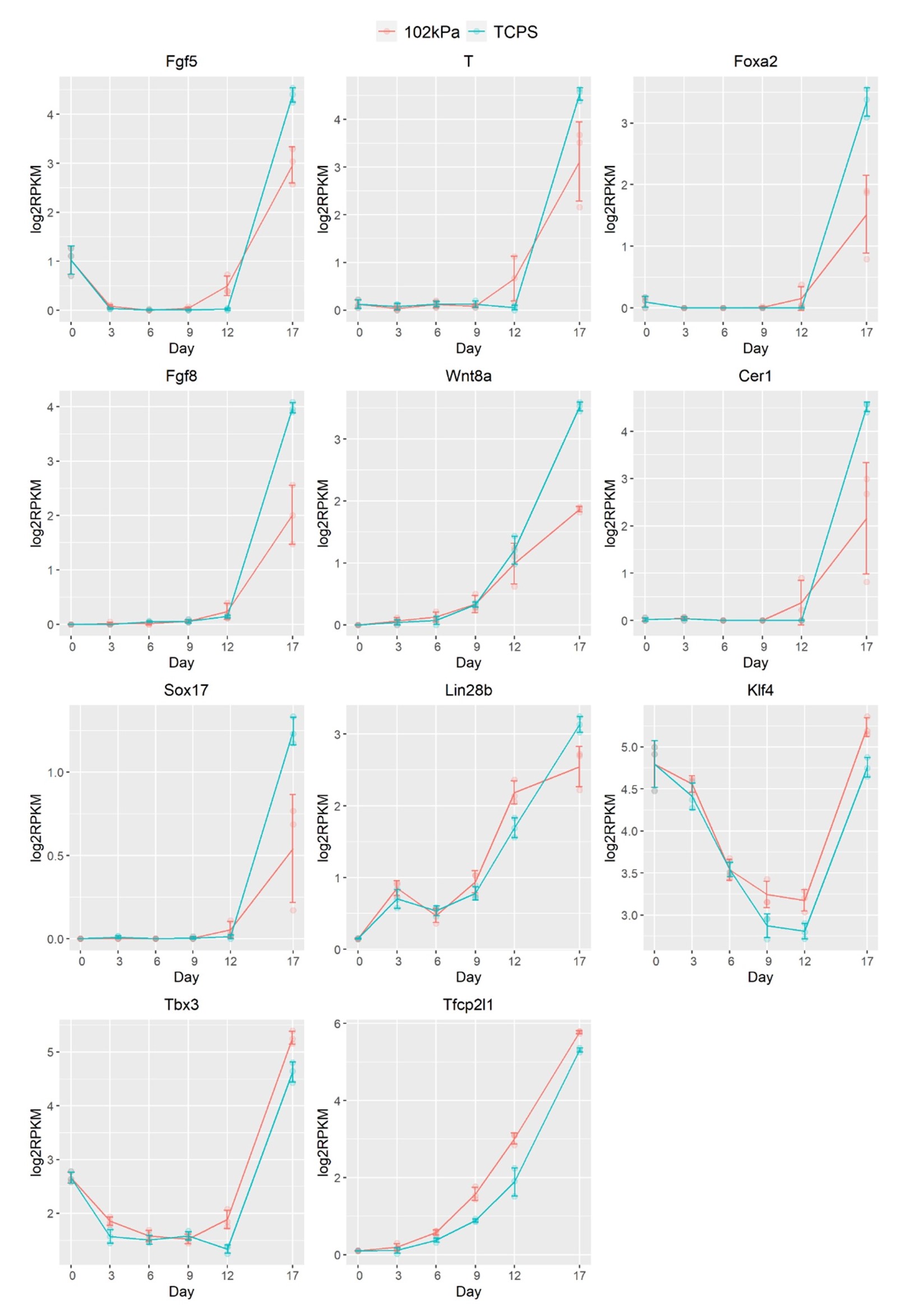

Supplement: Supplementary 1 — Figs. S1 to S11 [file bmr.0025.f1.zip › SuppFig7.jpg]

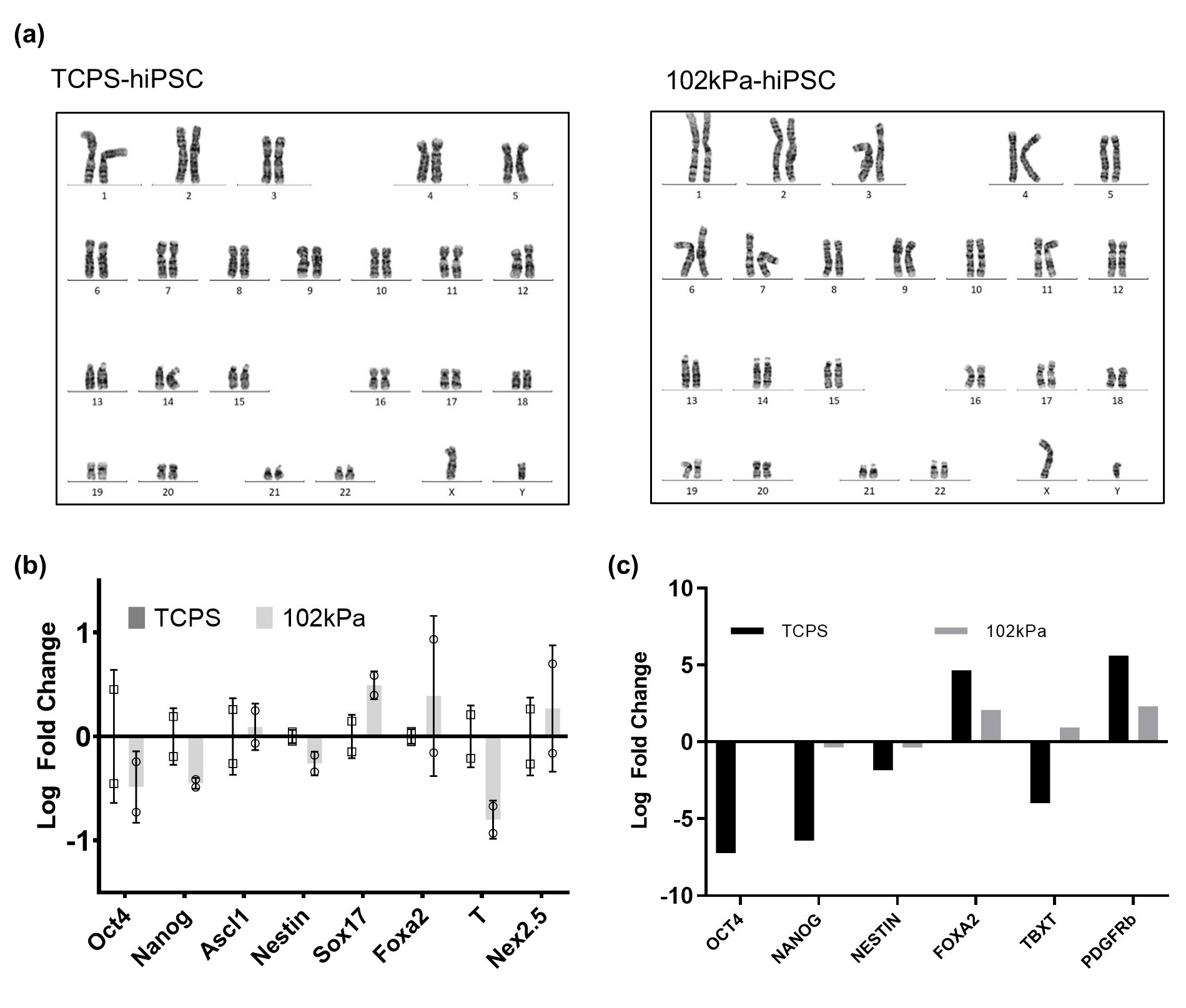

Supplement: Supplementary 1 — Figs. S1 to S11 [file bmr.0025.f1.zip › SuppFig8.jpg]

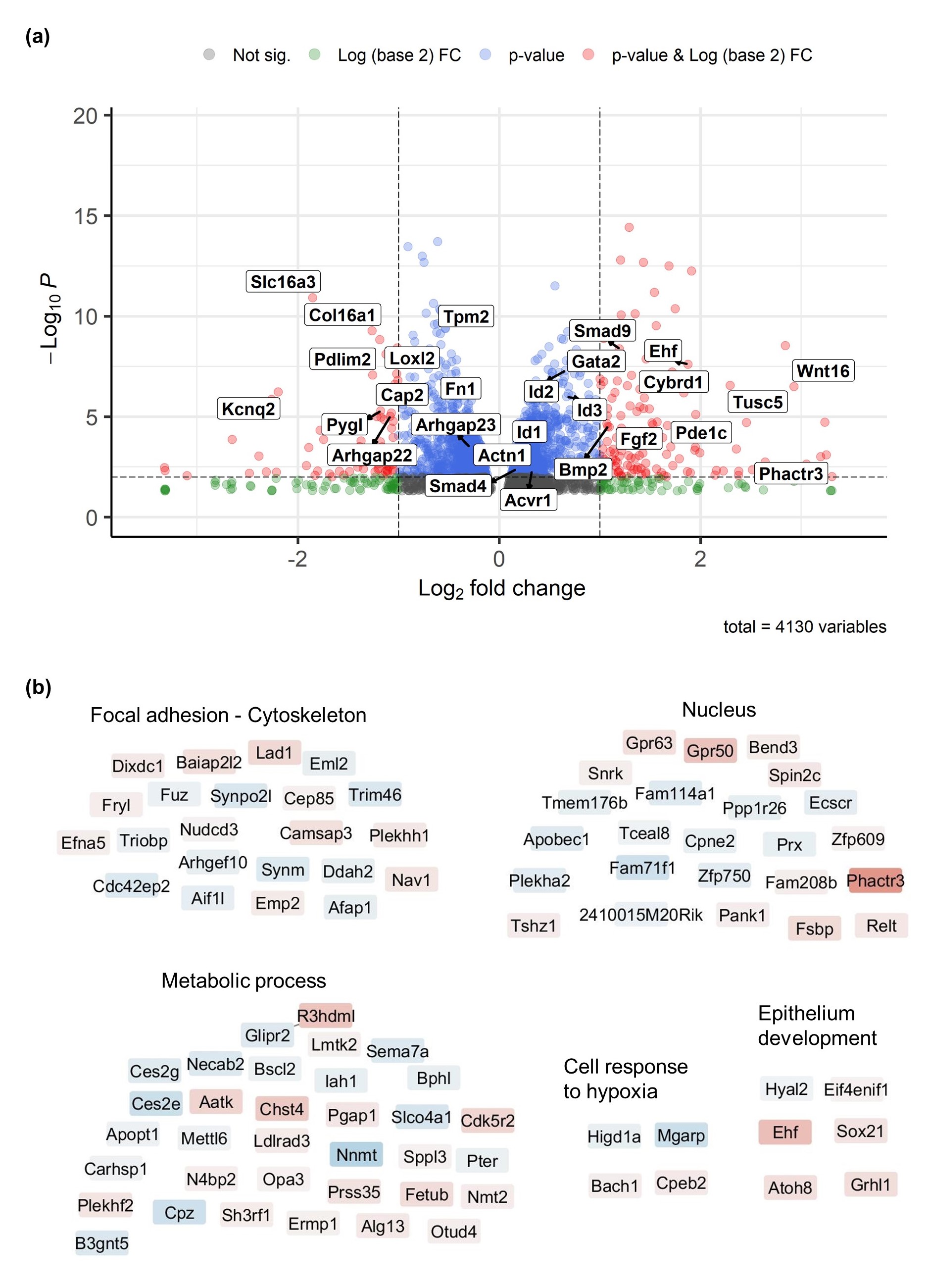

Supplement: Supplementary 1 — Figs. S1 to S11 [file bmr.0025.f1.zip › SuppFig9.jpg]
